# Targeting intracellular mRNA m^6^A-modifiers in advancing immunotherapeutics

**DOI:** 10.1016/j.jare.2025.06.030

**Published:** 2025-06-23

**Authors:** Sunil Kumar, Mithun Sinha

**Affiliations:** Department of Surgery, Indiana University School of Medicine, Indianapolis, IN, USA

**Keywords:** Epitranscriptomics, m^6^A-methylation, Cancer immunotherapy, RNA-modifying drug, Intracellular Immune checkpoints

## Abstract

An impressive clinical success has been achieved in the treatment of various cancers employing immunotherapy and checkpoint blockade antibodies. However, limited therapeutic response has been observed in most of the patients receiving anti-PD1 antibodies remains a critical challenge, underscoring the need for innovative strategies to enhance immunotherapeutic efficacy to overcome these barriers. Several epigenetic drugs targeting DNA and histone proteins have received FDA approval are in the market. However epigenetic drugs targeting mRNA-modification machineries are still in infancy. The discovery of RNA modification enzymes (writers, erasers, and readers) has refuelled the scientific horizons in overcoming the challenges mainly associated with other pharmaceutical drugs. RNA modification especially ‘m^6^A’ is one of the well-studied epitranscriptomic mechanisms involved in regulating diverse biological processes *via* mRNA metabolism. To date more than 170 different types of RNA-modifications have been identified covering the different aspects of cellular, immunological, and biological functions. In this review we aim to summarize the potential of utilizing m^6^A-modifiers in discovering novel therapeutic procedure targeting immune cell subsets. Moreover, we have highlighted the possibilities of improving personalized therapy co-targeting m^6^A-modifiers engineered with other intracellular checkpoint genes like CISH. Furthermore, we have proposed dual therapeutic approach combining m^6^A-modifiers with anti-PD1 antibodies to enhance the efficacy of targeted medicines. Additionally, we have mentioned few biopharmaceutical companies and cell therapy clinics developing m^6^A-based RNA-modifying drugs (RMDs) and associated clinical trials. Our study will advance foundational knowledge in nurturing the potential of ‘RNA epigenetic modifiers’ in discovering novel therapeutic drug candidates, enhancing personalized therapy through immune cell engineering.

## Highlights

• Epitranscriptomics is the epigenetic study of RNA-modification without altering gene sequence.
• m^6^A, a key mRNA modification among 172 types, is regulated by Writers, Erasers, and Readers to control mRNA fate.
• m^6^A-modification modulate immunological functions in different immune cell subsets.
• Histone modification (H3K36me3) and microRNAs can also regulate m^6^A-modification through crosstalk and feedback mechanisms.
• Co-targeting m^6^A-modifiers boosts immune cell therapy and could overcome anti-PD1 checkpoint blockade resistance.

## Graphical abstract

**Figure:**
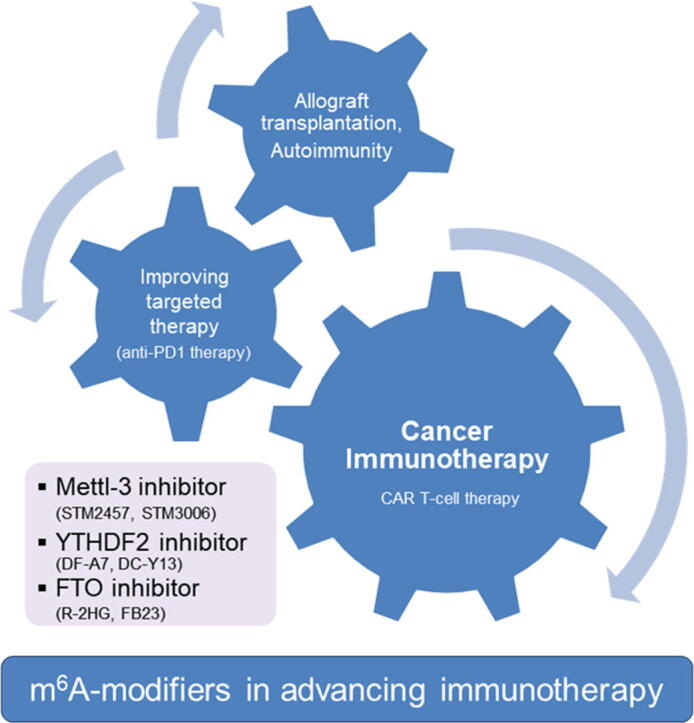

## Introduction

Epigenetic alterations have contributed greatly to human carcinogenesis. Conventional epigenetic studies have predominantly focused on DNA methylation, histone modifications, and chromatin remodelling [[1], [2], [3]]. All biological molecules such as DNA, RNA, proteins, lipids and sugars are subjected to covalent modification, which are highly specific, resulting in a wide range of biological functions. However, RNA modification has recently emerged as a new layer of epigenetic regulation and revolutionized the field of clinical and therapeutic research. The term “Epitranscriptomics” refers to the study of chemical modifications on RNA molecules, particularly mRNA, which regulate gene expression without altering the underlying genetic code [4,5].

Epigenetic modification is a chemical modification of DNA, RNA and histone proteins. These external modifications elicit a broad spectrum of biological and physiological alterations without perturbing the primary backbone sequences, thereby garnering substantial attention in recent times. To date, DNA and histone protein modifications have been extensively investigated, with several epigenetic drugs already available on the market [[6], [7], [8]]. However, RNA modifications have recently re-emerged as a focal point, reigniting both basic and clinical research, particularly in the areas of cancer biology and immunotherapy. In this review, we have focused on the impact of RNA modifiers, especially methyl-6 Adenosine (m^6^A)-modification in improving immunotherapeutics. In addition, we have also shed light on the application of m^6^A-modifiers in enhancing the efficacy of standard targeted therapies and treating various clinical diseases by developing RNA modifying drugs (RMDs) as viable drug candidates.

### Discovery of RNA epigenetics (Epitranscriptomics)

The discovery of epigenetic drugs targeting DNA and histone modifications dates to 1960 s [9,10]. However, the term “epigenetics” was first coined in 1942 by Conrad Waddington [11]. On the contrary the term “epitranscriptomics” which deals with mRNA modification was recently coined by Dr. Chuan He in 2010 [12]. The first mRNA modification-based epigenetic drugs m^6^A-demethylase fat mass and obesity (FTO) was discovered by Dr. He et al., in 2011 [13] which refuelled the passion in m^6^A research because of its dynamic, reversible and post-transcriptional modification abilities [14]. Now a series of mRNA-based drugs is currently under clinical trials targeting m^6^A-modification machineries [15].

### Why epigenetic modification of RNA is important?

**Fig. 1:**
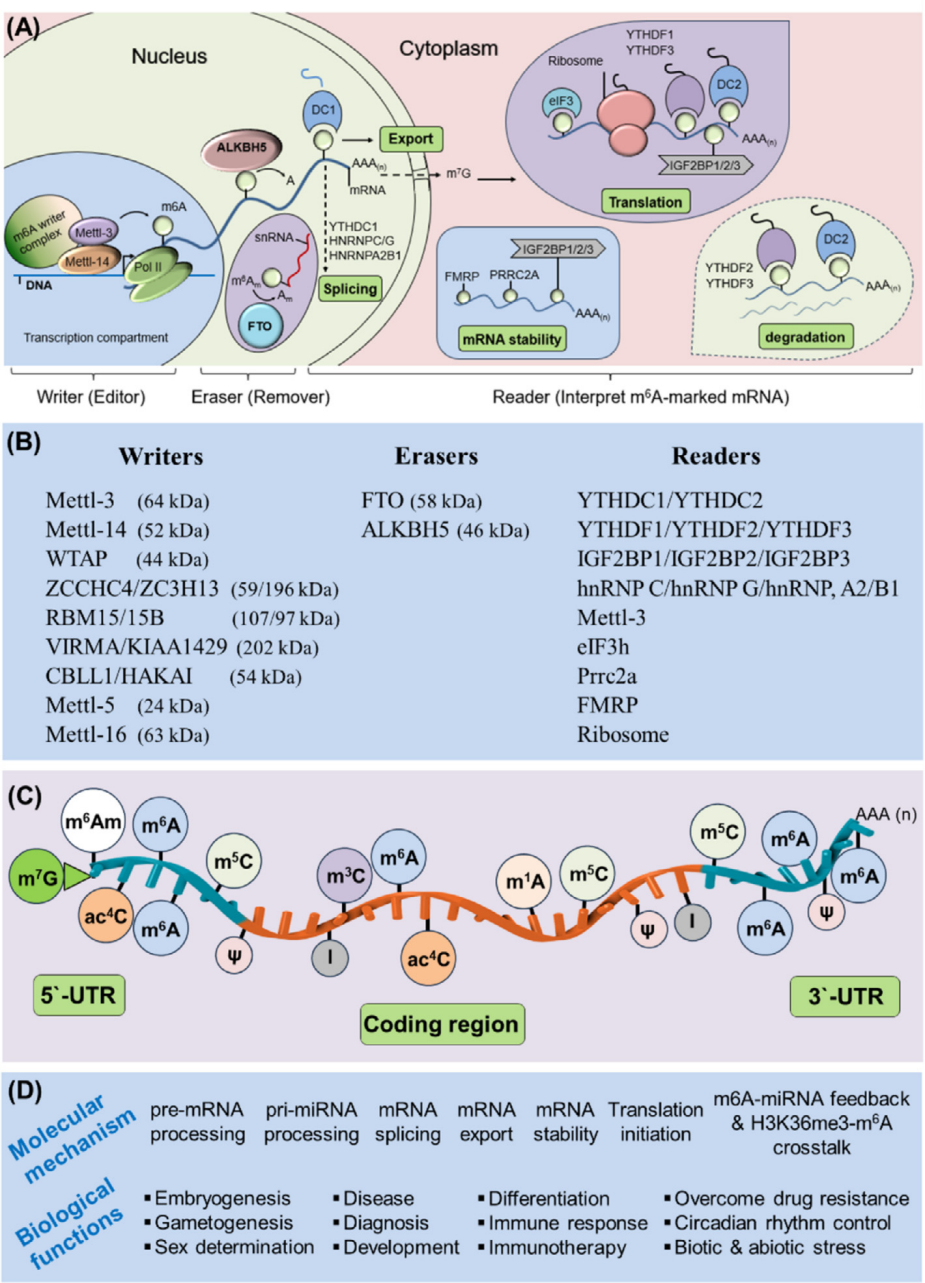
Epitranscriptomic biology of m^6^A-modified mRNA: **A** Biogenesis of m^6^A-modified mRNA, include: (i) microRNA processing by HNRNPA2B1, (ii) alternative splicing by YTHDC1, HNRNPC/G and HNRNPA2B1, (iii) nuclear export by YTHDC1, (iv) translation by YTHDF1, YTHDF3, YTHDC2, IGF2BP1/2/3 and eIF3, (v) mRNA stability by IGF2BP1/2/3, FMRP & PRRC2A, and (vi) mRNA degradation by YTHDF2, YTHDF3 and YTHDC2 proteins. However, the current hypothesis states that all three YTHDF1, YTHDF2 and YTHDF3 are localized in cytoplasm and are involved in mRNA degradation. **B** The major mRNA m^6^A-modification enzymes/proteins (RME/RMP). **C** Possible types of mRNA modification. **D** Molecular mechanism and biological role of m^6^A-modified mRNA.

Despite DNA and proteins being highly decorated with various modifications, recently most attention has been directed towards RNA modification because of its unique features comparative to the conventional DNA and histone protein-based modifications [16]. For example, RNA is positioned in the nucleus as well as cytoplasm and thus regulating major cellular processes (Fig. 1**A**). It is well established that RNA is fundamental to all living organisms. An average human cell consists of approximately 10–50 picograms (pg) of total RNA likely: ribosomal RNA (80–85 %), transfer RNA (10–15 %) and messenger RNA (2–5 %). However, only messenger RNA (mRNA) has got high attention because of its protein coding genes. It has been expected that there are more than 100 thousand distinct mRNA transcripts possible after alternative splicing from 20 to 25 thousand coding genes in a human genome [17]. Therefore, the abundance of these mRNA transcripts has sparked researchers' interest in studying different mRNA modifications and responsible enzymatic machineries (Fig. 1**B**) linked to diseases and immunological responses. Contemplating the structure of eukaryotic mRNA, it consists of five major components: 5′ cap, 5′-untranslated region (5′-UTR), coding region, 3′-untranslated region (3′-UTR) and poly-A tail. However, the most abundant chemical modification N^6^-methyladenosine (m^6^A) is found to be enriched in the vicinity of the stop codon at 3′-UTR [18], indicating the crucial role of m^6^A in regulating mRNA metabolism and thus impart various molecular and biological functions (Fig. 1**C and D**) [19]. To date approximately 172 different types of total RNA modifications have been identified in humans [20]. The consensus motif required for mRNA modification is DRACH (D = A, G or U; R = G or A; H = A, C, or U), where **R** is a purine (adenine or guanine), **A** is the methylated adenosine, and **H** is a non-guanine base (adenine, cytosine, or uracil) [21]. Among these 172-modifications in total RNA, the most abundant modification is m^6^A-modifications [17] while the other possible RNA modifications are: m^1^A, m^3^A, m^6^A_m_, m^5^C, ac^4^C, m^7^G, Inosine (I), Pseudouridine (ψ) etc. [4,20]. However, this review focuses more on the application of m^6^A-modifiers in advancing immunotherapeutics.

### Clinical significance of mRNA m^6^A-modification

The clinical significance of RNA epitranscriptomics is evidenced by various publications in the areas of fundamental biology and clinical research, highlighting its growing impact and relevance in improving immunotherapeutic scope. For example in (i) Basic molecular biology: (gene expression [22], mRNA stability [23,24], translation [25,26], miRNA processing [27], epigenetic research [28,29]). (ii) Developmental biology: (embryonic development [30,31], sex determination [32], mitosis/cell-cycle regulation [33], spermatogenesis [34], stem cell pluripotency [35], cerebellar development/neurogenesis [36,37]). (iii) Immunology & immunotherapy: (immune evasion [38,39], anti-cancer immunity [40,41], autoimmunity [42], allograft transplantation [43,44]). (iv) Cancer biology: (leukemogenesis [45,46], solid tumors [[47], [48], [49]], osteosarcoma [50]). (v) Cancer treatment [41,51]. (vi) Drug resistance [52,53], and (vii) other diseases like: (obesity [54], diabetes [55,56], rheumatoid arthritis [57]). In addition to these, our investigation adds the role of m^6^A-modifiers in (viii) mitigating CD4^+^T-cell pathogenicity, (ix) regulating autoimmunity, (x) prolonging organ transplantation, (xi) preventing the infiltration of immunosuppressive cells, (xii) increasing effector immune cell infiltration, (xiii) targeting intracellular immunecheckpoint genes (such as CISH, SOCS, and PD-1/PD-L1) in improving metabolic fitness, and (xiv) boosting the efficacy of anti-PD1 therapy. Collectively, involvement of these mRNA m^6^A-modification enzymes in variety of human diseases clearly suggests its clinical importance and as a therapeutic target in discovering novel drug candidates and better optimizing the personalized therapies.

## Life cycle of m^6^A-modified mRNA

The lifecycle of m^6^A-modification is categorised into three major stages: Writer, Eraser and Readers. ‘Writers’ catalyse m^6^A-installation, ‘Erasers’ remove the modification, and ‘Readers’ interpret m^6^A-marked RNA to regulate specific function [58]. The journey of m^6^A-mRNA initiates in the nucleus, there the major m^6^A-writer complex (Mettl-3 and its cofactor Mettl-14) methylate within the consensus ‘DRACH-motif’ at the 3′-UTR of the mRNA, which was transcribed from the respective DNA strand by RNA polymerase-II [59]. This phase is occurring in the transcriptional compartment therefore it is considered as “Writer” phase. Next, the “Eraser” phase is also occurring in the nucleus and is known to remove the methylated group by its demethylase activity. The major enzymes required for demethylation are ALKBH5 (AlkB homolog 5, RNA demethylase) and FTO (fat mass and obesity). ALKBH5 mainly acts on ‘m^6^A’ and removes ‘A’ from m6, whereas FTO preferentially target ‘m^6^A_m_’ present on small nuclear RNA (snRNA), (Fig. 1**A**). Finally, the “Reader” phase which appears to function in both nucleus as well as in the cytoplasm. In the nucleus YTHDC1 (DC1) binds to the methylated m^6^A and facilitates ‘splicing’ as well as ‘export’ of the mRNA. In the cytoplasm the other reader proteins like YTHDF1, YTHDF3, eIF3 and Mettl-3 binds to the marked m^6^A and favour ‘translation’ of m^6^A-modified mRNA. Whereas YTHDF2, YTHDF3 mediates ‘degradation’ of m^6^A-mRNA, while IGF2BP1/2/3 (Insulin-like growth factor 2 mRNA-binding proteins) and FMRP (a polyribosome-associated RNA-binding protein) enhances the ‘stability’ of the m^6^A-modified mRNA [4,5,12]^.^ Furthermore, the average half-life of human mRNA is approximately 10 h, but varying with the length of the cell-cycle and other transcription factors [60]. However, m^6^A-modification generally confer ‘mRNA-instability’ suggesting that m^6^A-modified mRNA have shorter half-life than the unmodified mRNA [17,59]^.^ Similarly, the stability of m^6^A-modified mRNA is also affected by several factor like the recruitment of YTHDFs proteins, number of m^6^A sites on the mRNA and the specific location of m^6^A installations, or weather at 5‘-UTR, coding region or at the 3‘-UTR of the mRNA [61,62].

### Regulation m^6^A-modified mRNA

The major epigenetic enzymatic enzymes required for DNA modifications are methylases (DNMTs) which add methyl (CH_3_) group to the CpG island of the DNA, and de-methylases (TETs) that removes the methyl group from that DNA strand [[63], [64], [65]]. Similarly, for histone protein modifications acetyltransferases (HATs) are required which add acetyl (C_2_H_3_O) group to the histones and deacetylases (HDACs) that remove acetyl group from the same [8,66]. Likewise, for RNA modifications the major epitranscriptomic enzymes required are Writers (methyltransferases) which add methyl and other group to the mRNA, Erasers (de-methylases) which removes methylated group and Readers that interpret m^6^A-marked mRNA for further processing [4,67]. The details of mRNA modification machineries and its functions are discussed below.

#### Writers (Editors)

RNA-methyltransferase are the main enzymes responsible for the installation of ‘m^6^A’ on poly (A) mRNA [68,69]. It adds methyl group to the 6th position of adenosine (N6-methyladenosine or m^6^A) via adenosine methyltransferase, using S-Adenosyl methionine (SAM) as the methyl donor, and so it is called m^6^A-methylation or modification [70]. This is also known as ‘writers’ or ‘editors’ because of its editing or installation property. The corresponding homolog in plants is known as ‘MTA’ and in *drosophila* as ‘Ime4′ (Inducer of meiosis 4) in yeast [71]. *Significance*: The importance of methyltransferases was verified by the deletion of Mettl-3 or CRISPR-mediated inactivation of its allosteric adaptor/subunit Mettl-14 in the loss of more than 99 % of m^6^A in the mRNA of mouse ES cells [72]. *Structure*: The total size of methyltransferase complex is > 1MDa, where the smaller complex methyltransferase A (MT-A) contains Mettl-3 (580 AA, 64 kDa) and Mettl-14 (456 AA, 52 kDa) [21,73,74], while the larger complex methyltransferase B (MT-B) contains additional subunits like WTAP (44 kDa), ZC3H13 (196 kDa), RBM15/15B (107/97 kDa), VIRMA/KIAA1429 (202 kDa) and CBLL1/HAKAI (54 kDa) [74,75] (Fig. 1**B**). *Localization*: it is localized in both nucleus as well as in the cytoplasm suggesting its role in both transcription and gene regulation [5,76]. A consensus motif in the mRNA sequence DRACH (D = A/G/U; R = A/G; H = A/C/U) is required for m^6^A installation [21,77]. This appears once in every ∼ 57 nucleotides in the mRNA, however, not all DRACH site has potential to methylate [[78], [79], [80]]. *Function:* m^6^A-methylation mainly enriched near start codon 5‘-UTR [81], stop codon 3‘-UTR [18,82] and long internal coding regions (exons) [83] suggesting its role in transcription as well as in gene regulation [4,59]. *Dysregulation*: the dysregulation of Mettl-3 writer complex has been found to be associated with disruption in molecular mechanisms linked with several diseases and cancer [84,85]. *Mechanism*: the molecular mechanism for this site-specific m^6^A installation is poorly understood however, there are 4-major mechanisms proposed for m^6^A installation [4,59] are (i) transcription factor mediated methylation: for example, the methylation of SMAD2/3 transcript by the recruitment of m^6^A writer complex to the promoter by SMAD2/3 transcription factors in response to TGFβ signalling [86], in addition, the number of CpG island and structure of the promoter also influences mRNA m^6^A-methylation [87], (ii) histone H3-mediated methylation: this is confirmed by knockdown of the SETD2 (SET domain containing 2, histone lysine methyltransferase) which is responsible for H3K36me3 formation, resulted in a 40 % reduction in m^6^A levels in mRNA [29], (iii) RNA binding protein-mediated methylation: the recruitment of RNA binding domains (RBM15/15B) to m^6^A writer complex facilitates m^6^A methylation [5], and (iv) RNA polymerase-II mediated methylation: this is confirmed by slowing the process of RNA polymerase-II by using RNA Pol-II mutant or specific inhibitors in increasing the level of m^6^A methylation [87]. *Therapeutic implications*: pharmacological inhibitors of Mettl-3 and its therapeutic implications are mentioned in Table 1, Table 3.

**Table 1:** Immune cells targeting epitranscriptomic machineries in advancing personalised therapy.

**Immune Cells**	**Disease/** **Immune Role**	**Modification/ Mechanism**	**Target gene/** **Function**	**Ref.**
**T-cell subsets***(Adoptive Immunity)*				
CD4^+^T-cells	T-cell homeostasis (Colitis)	Mettl-3 (*slower mRNA decay*)	IL-7/Stat5 signalling SOCS-1, SOCS-3, **CISH**	[130,131]
CD4^+^T-cells	T-reg differentiation, dysfunction (Colitis)	Mettl-14	Th1, Th17 cytokines	[132]
CD4^+^T-cells	T-cell pathogenicity (SLE, EAE model)	ALKBH5 (*decrease mRNA stability*)	Decreased IFNγ and CXCL2, reduces CD4^+^T-cell pathogenicity	[135]
CD4^+^T-cells and PBMCs	Autoimmunity (SLE)	ALKBH5, Mettl-14, Ythdf2	Increased anti-dsDNA, C3/C4-complement level, apoptosis but decreased T-cell proliferation	[136,137]
CD4^+^T-cells	T-cell activation (SLE)	Mettl-3 (STM2457) (*mRNA decay*)	Foxp3 mRNA, Regulatory T-cells	[133]
CD4^+^T-cells	T-cell effector function (allograft transplantation)	Mettl-3 (STM2457)	(TFs: Ki-67, T-bet, c-Myc) Increased T-cells effector function cause allograft rejection	[134]
CD8^+^T-cells	Anti-tumour activity (TNBC, HCC)	Mettl-3 inhibitor + anti-PD1	IFN-γ, CXCL10, MHC-I, PD-L1	[[138], [139], [140]]
Ƴδ T-cells	Mucosal Immunity	ALKBH5 (*mRNA decay*)	Jagged-1 mRNA (NOTCH signalling)	[225]
γδT1-cells	(Psoriasis) γδT-cell differentiation	Mettl-3 (*mRNA decay*)	Th-17 cytokines, STAT1-IFNγ signalling, dsRNA formation	[149]
T_FH_-cells	Humoral Immunity (B-cells →Ab prod.)	Mettl-3 + IGF2BP2 (*mRNA stability*)	Tcf-7 mRNA (TCF-1 gene) Bcl-6, Icos, Cxcr5, PD-1, **CISH**	[144]

**Regulatory T-cells (T-regs)**				
Regulatory T-cells (intratumoral T-regs)	Anti-tumour immunity (melanoma and CRC)	YTHDF2-mediated degradation of NIrc3, Nfkbie and Traf3	YTHDF2 inhibition (YTHDF2^cKO^) is good in increasing the ATI of T-regs by regulating NFκB pathways	[147]
Regulatory T-cells	T-reg suppressive function	Mettl-3	Mettl-3 retained T-reg suppressive function by inhibiting **CISH**/SOCS-3	[145]
Regulatory T-cells	Allograft transplant (Islets)	Mettl-14	Mettl-14 inhibition reduce T-reg suppressive function and cause allograft rejection by targeting **SOCS** proteins	[43]

**B-cell subsets**				
B-cells	B-cell development (B-cell transition)	Mettl-3/14, YTHDF2 (*mRNA decay*)	Large pre-B cell, small pre-B, Chromatin remodelling	[150]
B-cells (CD19^+^)	GC-response (P. cells →Ab prod.)	Mettl-14, YTHDF2 (*mRNA decay*)	Lax1 and Tipe2 (negative regulators of GC selection)	[153]
DLBCL (Pro B-cells)	Diffuse large B-cell lymphoma	Mettl-3 inhibitor	PEDF, Stage-specific B-cell development	[152,155]

**Natural Killer cells (NK-cells)**				
NK-cells	NK-cell activation and effector function (Lung metastasis)	Mettl-3, Mettl-14 IGF2BP1,3 (*mRNA stability*)	FasL, Perforin, Granzyme-B, mTOR signalling	[158]
NK-cells	Anti-tumour immunity	Mettl-3	SHP2 (suppress AKT, MAPK signalling)	[159]
NK/T-cells	Lymphoma (NKTCL)	WTAP, Mettl-3 (*mRNA stability*)	WTAP: DUSP6 mRNA Mettl-3: SND1 mRNA	[160,161]
NK-cells NK92 cell line	Lung metastasis, Leukemia	FTO, JAK/STAT, **CISH, SOCS1-3**	FTO negatively regulates the cytotoxic activity of NK-cells by targeting IFNγ, Perforin, Granzyme-B, TNFβ.	[162]
NK-cells	Anti-tumour immunity (B16F10-tumor)	YTHDF2 is required to increase anti-tumor immunity	Tardbp, NKG2D, IFNγ, Perforin, Granzyme-B. (Stat5-Ythdf2 + ve feedback loop)	[163]
NK-cells	NK-cell cytotoxicity against CRC	YTHDF2 is required to increase NK-cell cytotoxicity	SMAD4-YTHDF2 regulatory axis, NKG2D, Gzm-B	[164]

**Dendritic cells (DCs)***(Innate Immunity)*				
Dendritic cells	DC activation	Mettl-3 + YTHDF1 (*mRNA translation*)	CD40, CD80, Tirap adopter mediated TLR4/NFκB signalling	[165]
Dendritic cells	Anti-tumour immunity	Mettl-3 + YTHDF1 (*mRNA stability*) +anti-PD1 therapy	lysosomal-cathepsins mRNA (YTHDF1-inhibition is good to increase anti-tumor immunity)	[166,167]
Dendritic cells	Cardiac heart transplant	Mettl-3 (shMettl3)	(CD80/86, MHC-I/II, ICAM-1, IFNϒ, IL-12). Impaired DC-mediated T-cell inactivation prolong allograft survival	[169]

**Macrophage (MΦ)**				
Macrophage	Anti-inflammatory response	Mettl-3 (*mRNA stability*)	STAT-1 mRNA	[171]
Macrophage	Anti-tumour immunity (lung metastasis)	Mettl-3, YTHDF1 (*mRNA stability*) + anti-PD1 therapy	Spread 2 (ERK, NFκB, STAT3 signalling activation)	[173,226]
Macrophage	FTO, YTHDF2-med (*mRNA decay*)	STAT-1, STAT6, PPARγ (reduced NFκB-signalling)	siFTO increases Mettl3-med m^6^A-methylation and recruiting YTHDF2-mediated mRNA decay of macrophage target genes hampering its activation	[174]
Macrophage	FTO Type 2 diabetes	miR-495 inhibits FTO, promoting M1 MΦ polarization	miR-495/FTO axis	[56]
Macrophage	Mettl-3 (rheumatoid arthritis)	RA markers: CRP & ESR, andNFκB-signalling	Mettl-3 is significantly elevated & serves as a biomarker for RA	[172]
Macrophage	Inflammatory response	YTHDF2 (*mRNA decay*)	MAP2K4, MAP4K4 mRNA and NFκB MAPK signalling	[175]
Macrophage(BMDMs)	Colitis, pulmonary allergic inflammation	IGF2BP2 (*mRNA stability*)	IGF2BP2 is required to shift M1 to M2 macrophage by targeting TSC1 and PPARγ.	[176]

**Other Myeloid Cells** (TIMs, TAMs, MDSCs)				
Tumor-infiltrating myeloid cells (TIMs)	Anti-tumour immunity (CRC)	Mettl-3, YTHDF1-mediated (*mRNA translation*) of JAK1 mRNA, and H3K18 lactylation.	Mettl3-inhibition (Mettl3^f1/f1^LysM-cre/STM2457) is good to increase anti-tumor activity of TAMs	[142]
Tumor-associated macrophages (TAMs)	Anti-tumour immunity (CRC)	Mettl-14 is good as it is required to increase CD8^+^T-cell function	Mettl-14 inhibition in TAMs dysfunction CD8^+^T-cells and thus decrease ATI	[178]
TAMs	Anti-tumour immunity (melanoma and CRC)	Upregulated YTHDF2 in TAMs degrade STAT-1 mRNA	YTHDF2 inhibition increases CD8^+^Tcell-mediated anti-tumor immunity	[179]
MDSCs	Anti-tumour immunity (lung metastasis)	NF-κB signalling genes Tnfaip8l2, **SOCS3**, Smpdl3b, Metrnl, and Adrb2	YTHDF2 inhibition (Lyz^cre^YTHDF2^f1/f1^, DC-Y13) combined with radiation improve anti-PD1 efficacy	[180]

**Table 2:** Biopharmaceutical companies targeting m^6^A-modifiers as a druggable candidate and current clinical trials.

**Company**	**Target**	**Product**	**Disease**	**Clinical Trial**	**Ref.**
**Storm Therapeutics**UK, 2015	Mettl-3, Mettl-1 and ADARI	STC-15, BBB+/-ve 2nd Gen Mettl-3, Mettl-1 and ADARI	Solid tumors, AML, Alzheimer‘s	Pre-clinical & Phase-I NCT05584111	[143,209,211]
**Accent Therapeutics**USA 2017	DHX9 inhibitor, KIF18A inhibitor. Mettl3, ADAR1	ATX-559 (an oral inhibitor of DHX), KIF18A inhibitor	TNBC, AML, Solid tumors	Phase-1 NCT06625515	accenttx.com
**Gotham Therapeutics/ 8five8 Thera.**2014 USA	PARG, Mettl-3, Mettl-14, QPCT/L, ADAR1	PARG (ETX-19477), QPCT/L, ADAR1 and Mettl-3 inhibitors	Solid tumors, haematological cancers	Pre-clinical & Phase-I NCT06395519	8five8tx.com
**Epics Therapeutics**Belgium, 2018	Mettl-3 inhibitor	EP102, EP282, ESN-196 (CCR5 agonist)	Solid tumors, Gastrointestinal infla. disorders, AML, HIV	Discovery stage & pre-clinical	[214]
**28/7 Therapeutics, Redona Therapeutics** USA, 2016	ncRNA: Lin28/let-7 pathway, CLK, cGAS, TUT4/TUT7, PAPD5/PAPD7	Lin28 inhibitor, Terminal uridylyl transferase (TUTase targeting)	Solid tumors, Autoimmunity, Telomere disease, HBV	Discovery stage	https://www.twentyeight-seven.com
**H3 biomedicine**USA, 2011	SF3b (RNA splicing complex)	H3B-8800	AML, CML, MDS	Phase-I NCT02841540	[215,216,227]
**Ribometrix Therapeutics**USA, 2018	c-MYC RNA, eIF4E	c-MYC, eIF4E, Regnase-1	Solid tumors	Discovery stage	[217]
**Skyhawk Therapeutics**USA, 2018	Small molecule targeting RNA splicing factor	SKY-0515	Cancer, HD, rare genetic disease	Phase-IACTRN12624000602527	www.skyhawktx.Com
**Korro Bio**USA, 2014	Serpin-A1 gene mutation	AATD, ADAR1(OPERA technology)	liver and central nervous system	Discovery stage	https://www.korrobio.com [218]
**Wave Life Sciences**USA, 2012	RNA editing, splicing	**WVE-006** (AATD) Serpin-A1 gene	Lung and liver disease (G-to-A point mutation)	Phase-INCT06405633	https://www.wavelifesciences.com [219]
**ProQR Therapeutics**, Netherlands, 2012	Axiomer™ ADAR RNA editing	**AX-0810**: for NTCP, **AX-1412**: for B4GALT1	Cholestatic liver (SLC10A1 gene), and CVD	Proposed clinical trial by 2025	https://www.proqr.com

**Table 3:** Pharmacological inhibitors of m^6^A-modification enzymes.

**m^6^A-modifiers** **(Inhibitors)**	**Biological role**	**Target gene/cells** **and function**	**Biological function**	**Cell type /** **disease**	**PMID**
**Writers: Mettl-3 inhibitors or activators**					
STM2457 (STC-15)	Mettl-3 Inhibitor (*Competitive inhibitor*)	Binding & inhibition to Mettl-3 complex	Anti-tumor	AML cell lines	33,902,106 37,670,178
STM3006		dsRNA formation & Interferon response	Anti-tumor	CD8^+^T-cells	37,548,590 37,586,322
Cpb-564		IGF2BP2-med stability of TAB3 [TGF-β-activated kinase 1 (MAP3K7) binding protein 3]	Renal injury and inflammation	Tubular epithelial cells	35,417,191
UZH1a		Reduced the m^6^A/A ratio in mRNAs	Anti-leukaemia	AML: MOLM013 Osteosarcoma: U2OS cell lines, HEK293T	34,237,194 36,692,498
Adenosines derivatives and analogues		−	Anti-leukaemia	AML NCT01684150	32,159,918
SANCDB0370 SANCDB0867 SANCDB1033		derived from natural compounds	Anti-cancer	−	36,995,499
Chidamide		c-MET/ALK/ROS1	Anti-tumor	Non-small Cell Lung Cancer	32,792,859
Quercetin		derived from natural products, inhibits tumour cell viability	Liver, pancreatic and cervical cancer	MIA PaCa-2 and Huh7 cell lines	35,571,106
Simvastatin		EZH2 mRNA	Anti-tumor	Lung cancer	32,373,962
CDIBA	Mettl-3 Inhibitor (*Allosteric Inhi*.)	antiproliferative impact on multiple AML cell lines	Anti-proliferative	AML	35,040,501
Eltrombopag		anti-proliferative effects on MOLM-13	Anti-proliferative	AML	35,455,436
Piperidine/Piperazine-derivatives	Mettl-3 Activators	METTL3-14-WTAP complex activator	cell differentiation, proliferation & death	HEK293 cells	30,917,327
Nocardia rubra cell wall skeleton (Nr-CWS)	Mettl-3 Activators	CDP-diacylglycerol synthase 2 (Cds2) mRNA	Increases angiogenesis in HUVECs	Diabetic wound healing	39,424,634
H3K18 lactylation	Mettl-3 Activators	YTHDF1-mediated (*mRNA translation*) of JAK1 mRNA	Mettl3-inhibition required to increase ATI of TAMs	Anti-tumour immunity in CRC by increasing Tumor-infiltrating myeloid cells (TAMs)	35,320,754

**Erasers: FTO and ALKBH5 inhibitors or activators**					
R-2HG (R-2-hydroxyglutarate)	FTO Inhibitor (*Competitive inhibitor*)	Inhibit FTO, MYC/ CEBPA, PFKP/LDHB mRNA	Inhibit cell prolif., viability, promote cell-cycle arrest & apoptosis	Leukaemia cells (Anti-leukaemia activity)	29,249,359 33,434,505
FB23 and FB23-2 (tricyclic benzoic acid)		ASB2/RARA, MYC/ CEBPA mRNA	Suppresses proliferation and promote diff. & apoptosis	AML cell lines, Xenotransplant mice	30,991,027
13a (tricyclic benzoic acid inhibitors analog)		Up-regulate ASB2, RARA and down-regulate MYC	Stronger anti-proliferative effect	AML cells, MONOMAC6-transplanted NSG mice	35,793,358
Baicalin hydrate (natural compound)		Sat2, α-Sat, major-Sat, IKKα, Suv39H1, and H3K9me3	Anti-tumor	Nasopharyngeal carcinoma	29,416,665
CS1/CS2		LILRB4, MYC, CEBPA mRNA, Akt/ mTOR signalling	Attenuate leukaemia self-renewal, reprogram immune response, CS1 suppressed CRC cell proliferation	LSC/LIC cells, HCT116-5FUR cell line, colorectal cancer	32,531,268 32,559,428 36,874,096
1,2,3-triazole analogues C (compound C6)		EMT pathway and PI3K/AKT pathway	Antiproliferative activity against KYSE-150, KYSE-270, TE-1, KYSE-510, EC109 cell lines	Anti-tumor activity against Esophageal cancer	35,833,378
Saikosaponin A (SsA) Saikosaponin D (SsD)		FTO/m^6^A signalling, MerTK, Bcl-2	Suppress AML cell proliferation & promote apoptosis and cell-cycle arrest	AML	33,897,884
18077, 18,097		SOCS1 mRNA, P53 pathway	Inhibit proliferation and migration of cancer cells	MDA-MB-231 xenograft model, Breast cancer	35,256,950
CHTB, N-CDPCB		FTO-2 cell lines, Preadipocytes (3 T3-L1 cells)	decreased cell viability	Obesity-associated disease	26,915,401 26,314,339
Meclofenamic acid 2 (MA2), MA		Strongly inhibits growth and self-renewal of many cancer cell lines	PBT003-grafted mice showed reduced tumor and extended survival	(HeLa cells) Glioblastoma, Gefinitib-res. NSCLC cell line	25,452,335 35,530,301
MO-I-500		FTO and IRX3 proteins	Inhibit survival & colony formation of SUM149-MA cells	Triple-negative inflammatory breast cancer cell	27,390,851
Rhein (natural product)		Exhibits synergy with nilotinib or PKC412	Nilotinib and rhein significantly inhibits tumor growth	Leukemia	23,045,983
FTO-02, FTO-04, FTO-43		Anti-proliferative effects	Prevent neurosphere formation in patient-derived glioblastoma stem cells (GSCs)	Glioblastoma, AML, Gastric cancer	33,412,003 35,939,803
Quinolone derivatives		DNA gyrase and topoisomerase II	Anti-bacterial and anti-cancer activity	Neurodegenerative Parkinson diseases	33,926,120
GNPIPP12MA nanoparticles		Leukemia blasts, especially LSCs	Augments PD-L1 efficacy & increase CD8^+^T-cells	AML (Anti-leukaemia immunity)	35,119,204
Dac51 (a small-molecule compound)		c-Jun, JunB, and C/EBPβ	Restoring CD8^+^T-cell function in B16OVA tumor	Immune evasion, anti-tumor immunity, against melanoma	33,910,046
Entacapone (Comtan) *FDA approved in 1999*		FTO-FOXO1 regulatory axis	Induce apoptosis & cytotoxicity effect on esophageal cancer cell lines (KYSE-30, YM-1)	HepG2, ESCC cell lines. Esophageal and liver cancer	30,996,080 36,534,072
Imipramine (IMI) Amitriptyline (AMT) Tricyclic antidepressant	FTO activator	Stress-related neuropeptides	Increase FTO expression	Psychiatric diseases	34,527,794
2-[(1-hydroxy-2-oxo-2-phenylethyl)sulfanyl]acetic acid (3) and 4-{[(furan-2-yl)methyl]amino}-1,2-diazinane-3,6-dione (6)	ALKBH5 Inhibitor (*Competitive inhibitor*)	Suppress the proliferation of leukaemia cells	Anti-proliferative function	HL-60, CCRF-CEM, K562 Leukemia and Glioblastoma	34,056,479
ALK-04		Mct4/Slc16a3, tumour infiltrating Treg and MDSCs	Enhance anti-PD1 efficacy	Melanoma, Colorectal cancer	32,747,553
MV1035 (imidazobenzoxazin-5-thione)		CD73 protein	Reduce U87 glioblastoma cell line migration and invasiveness	Glioblastoma	31,937,477
Ena15, Ena21	ALKBH5 Inhibitor (*non-competitive inhibitor*)	FOXM1 mRNA	Inhibit cell proliferation	Glioblastoma multiforme	35,384,315
IOX1 (broad-spectrum inhibitor of 2-OG oxygenases)		ZDHHC3 mRNA, PD-L1	Anti-tumor	Glioma	36,566,230
20 m (1-aryl-1H-pyrazole scaffold)		FTO, ALKBH5	−	HepG2 cells	35,597,008

**Readers: YTHDF and IGF2BP1/2/3 inhibitors or activators**					
DF-A7 (IC_50_ = 53.83 nM)	YTHDF2 inhibitor	Sensitize CD8^+^T-cells & improve anti-PD1 therapy	Anti-cancer	Melanoma, NSCLC	38,820,140
DC-Y13 (IC_50_ 74.6 ± 1.9 µM)	YTHDF2 inhibitor	Inhibit MDSCs infiltration and improve anti-PD1 efficacy by targeting **SOCS-3**	Anti-cancer	Lung metastasis	37,236,197
Keratin type I cytoskeletal 17 (KRT17)	YTHDF2 degradation via ubiquitination	Enhance cytotoxic T-cell infiltration by targeting CXCL10 and improve anti-PD1 efficacy	Anti-cancer	Colorectal cancer	37,129,929
Cucurbitacin B (CuB)	IGF2BP1 Inhibitor (*Allosteric Inhi*.)	c-MYC mRNA	Anti-cancer	HCC	36,032,766
BTYNB		c-MYC, β-TrCP1 mRNA	Anti-cancer	Ovarian cancer, Melanoma, Leukemia, iCCA	28,846,937 36,816,728 32,761,127 36,736,530
Triptonide (Chinese herb Tripterygium wilfordii Hook)		Lnc-THOR-IGF2BP1 signalling	NPC cell cycle arrest, apoptosis activation	Nasopharyngeal carcinoma	30,503,558
7773		Kras mRNA	Anti-cancer	H1299, Es2, HEK293 cell line. Lung cancer	34,895,045
4-benzamidobenzoic acid class, and 6-reidothiophene class	IGF2BP2 Inhibitor (*Competitive inhibitor*)	Inhibits proliferation of tumour cells, HCT116, SW480, Huh7 cell lines	Anti-tumor	(Xenograft, zebrafish model), Colorectal & Liver cancer	35,023,719
JX5		IGF2BP2, NOTCH1, Jurkat cells	Anti-leukaemia	T-ALL xenograft model (leukaemia)	35,915,142
CWI1-2		IGF2BP2, KH4, glutamine metabolism pathway (Myc, GPT2, SLC1A5)	Anti-leukaemia	Acute myeloid leukaemia	36,306,790
Isoliquiritigenin (ISL) Chinese herb licorice	Inhibit IGF2BP3 expression (*No direct target*)	Stabilize TWIST1 mRNA	Repressed proliferation, migration, invasion	Non-small cell lung cancer	35,816,995
Berberine (isoquinoline alkaloids)	IGF2BP3	PI3K/AKT pathway	Inhibits proliferation and induces G0/G1 phase arrest	CRC	32,926,933
JQ1	IGF2BP3	ABCF1 mRNA	Post-transcriptional regulator of Ewing sarcoma malignancy	Ewing sarcoma	29,703,820
MO-460	hnRNPA2B1	Hypoxia-inducible factor-1α (HIF-1α)	Anti-cancer	Hep3B cells Hep3B	30,755,586

#### Erasers (Removers)

The m^6^A-eraser enzymes (also called m^6^A-demethylase) are the main enzyme responsible for the removal of m^6^A-methylation-mark on the RNA [88,89], mRNA (m^6^A) and snRNA (m^6^A_m_) [90]. The m^6^A-demethylase especially AlkB family consists of nine homologs, with the first eight designated as AlkBH1-8, and the ninth is known as FTO (fat mass and obesity-associated protein) [91]. *Importance*: FTO is the first m^6^A-demethylase discovered in 2011 demonstrating its efficient oxidative demethylase activity in-vitro on nuclear mRNA [92]. Some studies showed FTO has higher demethylase activity towards m^6^A mRNA, concluding the statement ‘m^6^A’ as a bona fide substrate for FTO [92]. However, some other studies indicate that FTO shows a non-specific interaction with m^6^A, as evidenced by the lack of a significant increase in m^6^A level following FTO knockdown in a mouse based on m^6^A transcriptome mapping. Additionally, FTO knockout in mouse embryos did not result in increased m^6^A level in mRNA [5,93]. However, some recent study showed FTO has substantially higher catalytic activity (approximately 100 times) for demethylating m^6^A_m_ rather than m^6^A [93] (Fig. 1**A**) confirmed by approximately 1,500 % increase in m^6^A_m_ level in FTO knockout cells as compared to the snRNA of wild type cells with < 5 % m^6^A_m_ via miCLIP [90]. Conclusively, FTO targets snRNAs indicating that it primarily regulates snRNA methylation rather than mRNA methylation. However, ALKBH5 has standard m^6^A-demethylase activity on mRNA. *Function:* despite m^6^A-removal, FTO has shown in mRNA splicing through m^6^A_m_-demethylation in snRNA [94], stability and translation [89,95]. *Structure*: The structure of FTO and ALKBH5 contains conserved 2OG-Fe(II)_oxy domains and coiled-coil domains, where FTO contains (505 aa) and ALKBH5 (394 aa) [73,96]. *Localization*: FTO has both nuclear (NLS) as well as cytoplasmic (Exportin-2) localization mediating protein shuttling between nucleus and cytoplasm [97]. FTO primarily target m^6^A in nuclear polyadenylated RNA in the cytoplasm (Fig. 1**A**). *Dysregulation*: FTO is highly expressed in certain cancer cells like AML and glioblastoma. Beyond its cellular expression in cancers, FTO is also abundant in various tissues, such as the brain, endocrine tissues (including insulin-producing pancreatic cells), adipose tissue, kidneys and liver. The dysregulation of eraser proteins has been shown in many cancer cases and obesity [89,96,98]. *Mechanism*: Despite the significant structural differences between the m^6^A demethylases FTO and ALKBH5, they also exhibit distinct mechanisms of action and regulatory roles. FTO utilizes a classical oxidative N-demethylation pathway, converting m^6^A →hm^6^A and gradually releasing adenosine (A) and formyl adenosine (FA). It regulates snRNA and m^6^A_m_ levels and mediates m^1^A demethylation in tRNA. Additionally, FTO demethylates m^6^A in mRNA, primarily targeting the m^6^A_m_ cap [99]. In contrast, ALKBH5 directly convert m^6^A →A and quickly releasing FA [88]. *Therapeutic implications*: some selective pharmacological inhibitors of m^6^A-eraser enzymes and its immunotherapeutic implications are mentioned in Table 1, Table 3.

#### Readers (m^6^A binding proteins)

The m^6^A-reader proteins are the main enzymes responsible for interpreting m^6^A marked-mRNA for further processing like; mRNA export, translation, stability and degradation. There are three major types of m^6^A-reader proteins (i) *directs readers*: these binds directly to the m^6^A modified mRNA, for example YTHDF1, YTHDF2, YTHDF3, YTHDC1, YTHDC2, eIF3, Mettl-3 and ribosome [61]. (ii) *indirect readers*: these indirectly binds to the bonafide m^6^A-binding proteins for example, IGF2BP1, IGF2BP2, IGF2BP3 & FMRP, and (iii) *m^6^A switch*: in this binding is controlled by structural changes triggered by m^6^A modification for example, HNRNPC, HNRNPG, HNRNPA2/B1 and Prrc2a [59]. *Importance*: The m^6^A-reader proteins are playing a crucial role in deciding the fate of the m^6^A-modified mRNA [61] and linked to various biological and molecular functions (Fig. 1**D**). *Structure*: YTH domain containing proteins were the first m^6^A-binding protein discovered in early 2010 s [77]. It contains conserved YTH-domains like structure [59,73]. YTHDF1 contains (559 aa), YTHDF2 (579 aa), YTHDF3 (585 aa), YTHDC1 (727 aa) and YTHDC2 (1430 aa). The details of other important domains & function are described well in these articles [100,101]. *Localization & function:* m^6^A-reader proteins are localized in both nucleus as well as in the cytoplasm and thus playing a crucial role in regulating various biological and molecular functions for example, (i) microRNA processing by HNRNPA2B1 [27,102,103], (ii) alternative splicing of mRNA by YTHDC1, HNRNPC/G and HNRNPA2B1 [104], (iii) mRNA nuclear export (nucleus →cytoplasm) by YTHDC1 [105], (iv) mRNA translation by YTHDF1, YTHDF3, YTHDC2, IGF2BP1/2/3 and eIF3 [26,106], (v) mRNA stability by IGF2BP1/2/3, FMRP & PRRC2A [23,107], and (vi) mRNA degradation (decay) by YTHDF2, YTHDF3 and YTHDC2 proteins [[108], [109], [110]]. However, the current model says all three m^6^A-reader proteins YTHDF1, YTHDF2 and YTHDF3 are localized in cytoplasm and are involved in mRNA degradation [59,80] (Fig. 1). *Dysregulation*: The dysregulation of m^6^A-reader proteins affects mRNA life cycle & metabolism and thus impair numerous cellular and physiological processes. The associated disease and several types of cancers linked with m^6^A-reader protein dysfunctions are discussed in these articles [58,101,111]. *Mechanism*: the detailed mechanism of m^6^A modifiers-mediated regulation of mRNA metabolism is discussed in section-2.2 below. *Therapeutic implications*: specific pharmacological inhibitors of m^6^A-reader proteins and its immunotherapeutic implications are mentioned in Table 1, Table 3.

### Molecular mechanism of m^6^A-modified mRNA

The regulatory function of m^6^A-modified RNA is driven by three major epitranscriptomic enzymes: writer, eraser and readers by metabolizing mRNA via (i) pre-mRNA processing, (ii) nucleus →cytoplasm export, (iii) mRNA translation, (iv) mRNA stability and (v) mRNA decay. **Pre-mRNA Processing** (*alternative splicing*): Alternative splicing is a fundamental mechanism in the maturation of mRNA from DNA, enabling a single gene to generate multiple protein isoforms and thereby contributes to diverse cellular and biological variations. This is evidenced by the loss of Mettl-3 in altered splicing patterns in various genes [112,113]. The major m^6^A reader proteins involved in mRNA splicing are YTHDC1, HNRNPC/G and HNRNPA2B1 (Fig. 1**D**) [85]. The multiple mechanisms involved in alternative splicing of m^6^A-methylated mRNA includes (i) recruitment of RNA-binding proteins (RBPs), for example YTHDC1 binds with SRSF3 and promote exon inclusion by displacing/inhibiting SRSF10 [104], (ii) modification of U6 snRNA catalysed by Mettl-16 [114,115], (iii) direct modulation of RNA-protein interactions, (iv) enrichment within exon and intron regions, (v) regulation of splicing factors, (vi) splice site recognition, and (vii) cooperation with other spliceosomal components [116]. **Nuclear Export:** m^6^A-methylated mRNA enhances its export from nucleus to cytoplasm, this was strengthened by deleting Mettl-3 in lowering mRNA export rate [117]. In contrast ALKBH5 deletion showed enhanced mRNA export into the cytoplasm [118]. An m^6^A-reader protein ‘YTHDC1′ has been shown in facilitating nuclear export [105]. **Translation**: YTHDF1, YTHDF3, YTHDC2, IGF2BP1/2/3 and eIF3 have been identified as key players in enhancing mRNA translation efficiency (Fig. 1**A**). The two main models proposed to explain the classical role of m^6^A-reader proteins in translation are (i) Mettl-3 (writer)-dependent pathway and (ii) m^6^A-reader dependent pathways. In *Mettl-3 dependent pathway*: Mettl-3 promotes translation by identifying m^6^A-methylated site at 5‘-UTR and 3‘-UTR via (a) cap-independent and (b) cap-independent pathways. In cap-dependent initiation of translation begins with the binding of eIF4F to m^7^G-cap at the 5‘-UTR [106]. However, in cap-independent pathways Mettl-3 binds to eIF3 and promote translation by forming a close-loop model [76]. In *YTHDF1-mediated pathways*: YTHDF1 binds to the 5‘-cap of methylated mRNA and thus enhance translation by recruiting translation initiation factors. In addition, YTHDF1 enhance methylated mRNA translation by binding to 3‘-UTR of m^6^A-modified mRNA which couple ribosomes to interact with eIF3b-subunit of the translation initiation complex [119]. YTHDF3 drives translation of m^6^A-rich transcripts in breast cancer brain metastasis [120]. YTHDC2 enhances translation through the RNA helicase [121]. **mRNA Stability**: m^6^A generally promotes mRNA degradation, though context-dependent exceptions exist [61,122]. IGF2BP proteins enhances mRNA stability and translation by recognising m^6^A methylated sites [123]. **mRNA decay** *(degradation)*: YTHDF2, YTHDF3 and YTHDC2 has been mainly found to be involved in mRNA degradation (Fig. 1**A**) [67,124]. YTHDF2 is a key decay-inducing reader protein. It degrades m^6^A mRNAs through two pathways. First, HRSP12 acts as an adaptor, linking YTHDF2 to RNase P or MRP for endoribonucleolytic cleavage when specific binding sites are present upstream and downstream. Second, YTHDF2 recruits the CCR4/NOT deadenylase complex, initiating mRNA degradation via exosomes and P bodies, which house decapping and exonuclease complexes like XRN1[109]. Additional evidence of YTHDF2 and YTHDF3 involvement in mRNA degradation includes: YTHDF2 degrade CDKN1B mRNA and accelerates cholangiocarcinoma progression [125]. YTHDF2 SUMOylation enhances mRNA degradation and cancer progression by strengthening its binding to m^6^A-modified mRNAs [110]. YTHDF3 enhances the translation and decay of m^6^A-modified RNA [108].

### Epitranscriptomic modifications & quantification methods

The m^6^A modification can occur in all three RNAs (t-RNA, rRNA and mRNA), however, m^6^A-mRNA modification is more important because of modification in the coding region and regulatory function. It has been found that approximately 1–2 % of the total adenosine is exposed for m^6^A-modification. Relatively, an average of 3–4 m^6^A-modification is possible per transcript. However, m^6^A percentage distribution has been found to be enriched near stop codon and 3‘-UTR than the other region.

#### RNA modifications

To date approximately 172 possible modifications has been identified in total RNA [17]. Among them N^6^-methyladenosine (m^6^A) modification has been found to be most abundant mRNA modification enriched near stop codon and 3‘-UTR of mRNA [18]. However, the other possible RNA modifications are; N^1^-methyladenosine (m^1^A), N^3^-methyladenosine (m^3^A), N^6^, 2‘-o- dimethyladenosine methyladenosine (m^6^A_m_), N^5^-methylcytidine (m^5^C), 5-hydroxymethylcytosine (hm^5^C), N^4^-acetylcytidine (ac^4^C), N^7^-methylguanosine triphosphate (m^7^G), Inosine (I) and Pseudouridine (ψ). Some additional modifications are 2′-O-methylation (2′-O-Me), 8-oxo-7,8-dihydroguanosine (8-oxoG), 5-methyluridine (m^5^U) and 2-thiouridine (s2U) [4,58] (Fig. 1**C**). These modifications are particularly regulated by (i) ubiquitination, (ii) SUMOylation, (iii) acetylation, (iv) methylation, (v) phosphorylation, and (vi) lactylation or noncoding RNA were described in these publications [28,110].

#### m^6^A quantification methods

mRNA m^6^A-quantification is very crucial to identify its distribution in the mRNA. There are several methods available to detect and quantify m^6^A-methylation [5,84] for example, (i) *Antibody-based methods*: (a) MeRIP-seq (Methylated RNA Immunoprecipitation Sequencing)[126]. (b) miCLIP (m^6^A individual-nucleotide resolution UV crosslinking and immunoprecipitation) [127], and (c) m^6^A ELISA [128]. (ii) *Chemical-based methods*: (a) m^6^A-REF-seq/MAZTER-sequencing and (b) DART-sequencing [126]. (iii) *Sequencing-based methods*: (a) direct RNA sequencing (DRS) and (b) nanopore RNA sequencing [126]. (iv) *Mass spectrometry-based methods*: m^6^A-LAIC-seq (m^6^A-level and isoform characterization sequencing) [126]. (v) *Enzyme-based methods*: SELECT (single-base elongation and ligation-based qPCR amplification method) [127], and (vi) *Computational methods*: m^6^Anet, m^6^Aboost, HOMER, MACS, and MeTDiff [129]. Each of these methods has its own advantages and limitations in terms of resolution, specificity and quantification capabilities.

## Immunotherapeutic strategies: targeting immune cells

There are two therapeutic strategies currently being utilized by the biopharmaceutical companies and research institutions to address various oncological and non-oncological conditions through the development of drugs targeting m^6^A-mRNA modification enzymes or proteins (RME/RMP). The first approach involves the administration of small molecules (oral or injectable formulation) that reprogram cellular RNA in cancer cells. These small molecules influence RNA modifications such as m^6^A-methylation, and thus impacts gene expression and protein synthesis in tumor cells. The second approach focuses on intracellular targeting of RNA-modifying enzymes in immune cells like, NK-cells, T-cells, regulatory T-cells, dendritic cells, macrophages and hematopoietic stem cells in context with adoptive transfer therapies. Both methods aim to reprogram cellular function through the modulation of RNA metabolism (RNA export, splicing, transcription, degradation) in treating various cancer types and other diseases. In this section we will provides an update on recent advancements in RNA biology, highlighting the emerging role of m^6^A modification as a promising drug target and its potential in immunotherapy as a personalized treatment strategy Table 1.

## T-cell subsets

### CD4^+^T-cells targeting Mettl-3 *(homeostasis)*

**Fig. 2:**
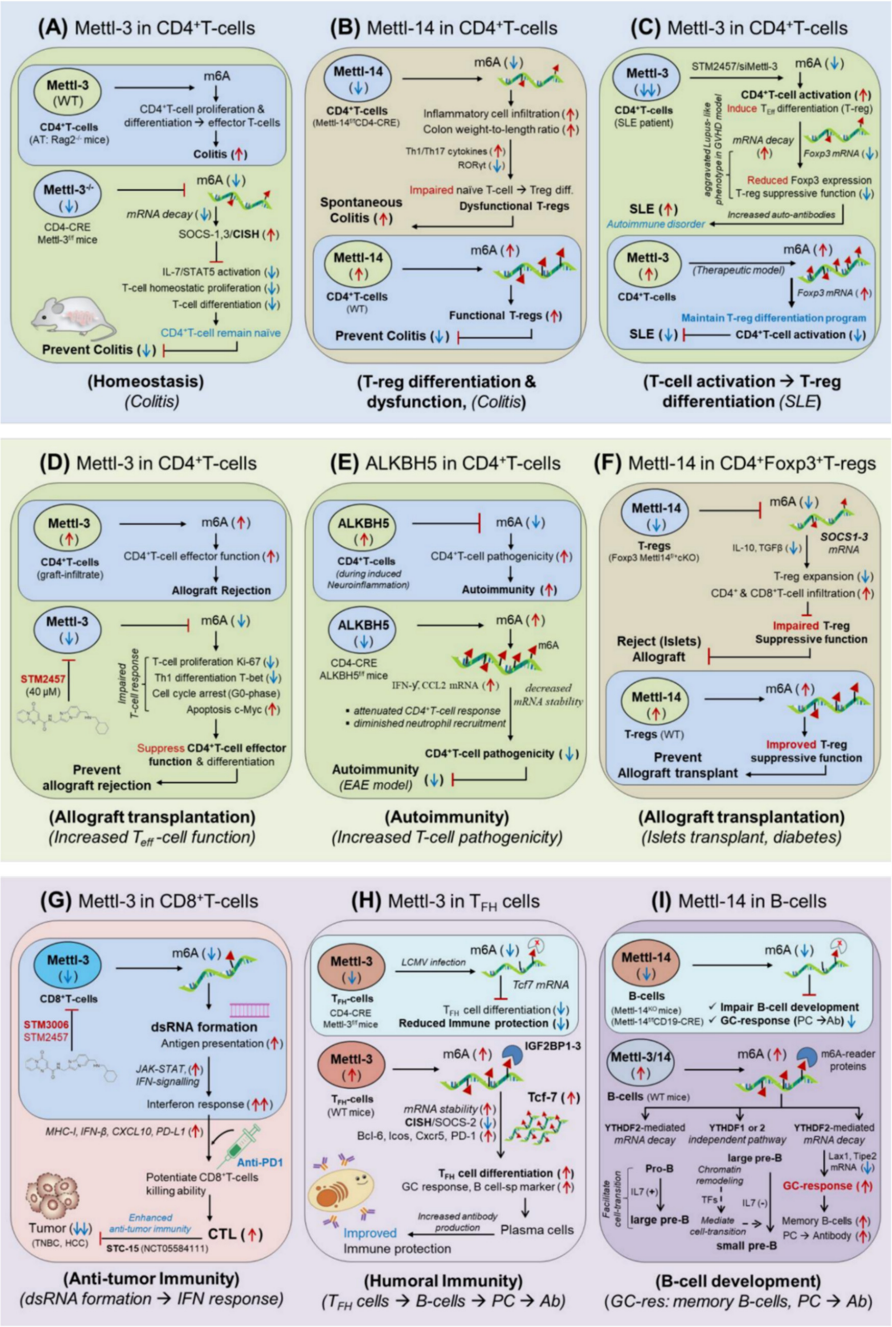
m^6^A-modifiers in regulating the immunological function of T-cell subsets. **A***CD4^+^T-cells in homeostasis (Colitis)*: CD4^+^T-cells expressing Mettl-3 facilitates T-cell proliferation & differentiation into effector populations causing colitis to develop. However, inhibiting Mettl-3 (Mettl-3^f/f^CD4-CRE) prevents colitis by keeping the cell under naïve state, implicating its therapeutic role by maintaining CD4^+^T-cell homeostasis. **B***CD4^+^T-cells in T-reg differentiation (Colitis)*: Mettl14-deficiency (Mettl-14^f/f^CD4-CRE) in CD4^+^T-cells facilitates the differentiation of dysfunctional T-reg cells, resulting in spontaneous colitis. However, Mettl14-mediated m^6^A modification control colitis by improving T-reg suppressive function by targeting Th-17 cytokines. **C***Mettl-3 in CD4^+^T-cell activation (SLE)*: Mettl-3 is drastically reduced in CD4^+^T-cells resulting in increased CD4^+^T-cell activation and impaired T-reg suppressive function causing SLE to develop. However, CD4^+^T-cell expressing Mettl-3 reduced SLE by lowering CD4^+^T-cell activation via m^6^A-mediated stabilization of Foxp3 mRNA. **D***CD4^+^T-cells in transplantation (allograft prevention)*: The major cause of allograft rejection is hyperactivation of CD4^+^T-cells effector program by upregulated expression of Mettl-3. However, pharmacological inhibition of Mettl-3 (STM2457) prevents allograft rejection by reducing the effector function of CD4^+^T-cells. **E***CD4^+^T-cells in autoimmunity (EAE model)*: m^6^A-eraser enzyme ‘ALKBH5′ is highly up-regulated in CD4^+^T-cells making it pathogenic resulting in increased autoimmune disease. However, specific deletion of ALKBH5 in CD4^+^T-cells (ALKBH5^f/f^CD4-CRE) reduces pathogenicity and decreased autoimmune response. **F***CD4^+^Foxp3^+^T-regs in Islets transplantation (diabetes)*: Mettl14-deficiency (Foxp3 Mettl-14^f/+^cKO) facilitates the proliferation of dysfunctional T-reg population resulting in allograft rejection. However, Mettl14-mediated m^6^A modification prolong islets survival by improving T-reg suppressive function by targeting SOCS1-3 family of proteins. **G***CD8^+^T-cells in anti-tumor immunity (therapeutic model)*: Mettl3-expressing CD8^+^T-cells showed impaired effector function. However, the pharmacological inhibition of Mettl-3 (STM3006, STM2457) promotes dsRNA formation and boost interferon signalling resulting in increased anti-tumor activity of CD8^+^T-cells. Moreover, it improved the efficacy of anti-PD1 therapy in dual therapeutic approach. A phase-I clinical trial (NCT05584111) targeting Mettl-3 by **STC-15** is scheduled against advanced malignancies by STORM therapeutics. **H***T_FH_ cells in (humoral immunity)*: Mettl3-deficiency (Mettl-3^f/f^ CD4-CRE) impair T_FH_-cells differentiation program resulting in decreased immune protection. However, Mettl3-engineered cells (retroviral overexpression) increased T_FH_-cell differentiation program resulting in improved immune protection by stabilizing Tcf7. **I***B-cells in (B-cell development, GC response)*: Mettl14-deficiency (Mettl-14^f/f^CD19-CRE) impair B-cell development and GC response. However, Mettl14-mediated m^6^A modification mediates B-cell transition and improves GC response via YTHDF2-dependent and Myc-independent pathways.

The study highlights the crucial role of Mettl-3 in modulating CD4^+^ T-cell in maintaining homeostasis [130]. To investigate the specific role of Mettl-3, the authors had generated conditional knockout mice by deleting Mettl-3 in CD4^+^ T-cells (Mettl-3^f/f^ CD4-CRE) and adoptively transferred to lymphopenic Rag2^-/-^ mice. 12 weeks later Mettl3-deficient CD4^+^T-cells failed to expand and lose their ability to differentiate into effector population and thus remain under naïve state (CD44^lo^CD62L^hi^) approximately 80 % as compared to the Mettl-3 ^wt^ mice (∼55 %). This prevented these mice from developing colitis and survived longer. Mechanistically, Mettl-3^f/f^ CD4-CRE mice showed the absence of both Mettl-3 & Mettl-14 and displayed overall decreased in m^6^A-methylation (72 %) relative to the wild-type CD4^+^T-cells. This caused slower ‘mRNA decay’ leading to increased expression of suppressor of cytokine (SOCS) family of proteins such as SOCS-1, SOCS-3 and CISH, a well-known negative regulator of cytokine signalling***.*** These elevated proteins suppressed IL-7/STAT5 signalling, essential for T-cell survival, however ERK/AKT signalling remained unaffected, which had supported T-cell survival by bypassing IL-7 signalling in Rag2^-/-^ mice. These findings demonstrate (i) SOCS-1, SOCS-3 and CISH are the direct target of Mettl-3 in CD4^+^T-cells, (ii) Mettl3-deficiency increases m^6^A-mediated expression of SOCS family of proteins which in turn inhibited IL7/STAT5 activation, T-cell proliferation & differentiation, and thus prevented from developing colitis. This work was further summarized and supported by *Jonathan et al.* in 2017 [131]^.^ Collectively, this result underscores the pivotal role of targeting Mettl-3 in maintaining in vivo T-cell homeostasis Fig. 2**A,** Table 1.

### CD4^+^T-cells targeting Mettl-14 *(T-regs differentiation/Colitis)*

This study highlights the clinical significance of Mettl14-expressing T-cells in preventing colitis, an inflammatory bowel disease [132]. The author showed that Mettl14-deletion in CD4^+^T-cells (Mettl-14^f/f^CD4-CRE) induces spontaneous colitis in mice, marked by increased inflammatory cell infiltration, colon weight-to-length ratio, Th1/Th17 cytokines, reduced RORγt expression, and impaired naïve T-cell induction into Tregs, leading to dysfunctional regulatory T-cells. Adoptive transfer of WT T-regs alleviated colitis and is dependent on the microbiome. This model provides a valuable tool for studying pathogenic pathways, microbiome contributions, and testing therapies for inflammatory bowel disease Fig. 2**B,** Table 1.

### CD4^+^T-cells targeting Mettl-3 *(T_eff_-cell activation/SLE)*

This study revealed the crucial role of Mettl-3 in the activation of CD4^+^T-cells in patients with systemic lupus erythematosus (SLE) [133]. Researchers observed that Mettl-3 is significantly reduced in CD4^+^T-cells derived from the peripheral blood of SLE patients compared to healthy controls. Moreover, pharmacological inhibition of Mettl-3 activity (STM2457) also supported the enhanced activation of CD4^+^T-cells (characterized by T_CM_: CD44^+^CD62L^+^ and T_EM_: CD44^+^CD62L^-^) phenotypes, while suppressed T-follicular helper differentiation (T_FH_: CXCR5^+^PD1^+^) and regulatory T-cells (T-reg: CD25^+^Foxp3^+^) in vivo. Additionally, this inhibition exacerbated lupus-like phenotypes in a graft-versus-host disease (GVHD) mouse model evidenced by increased serum levels of autoantibodies (IgG, dsDNA). Mechanistically, Mettl-3 silencing (siRNA) and STM2457 (Mettl3-inhibitor) treatment reduced the T-reg cell population by decreasing Foxp3 expression, mediated through reduced m^6^A-dependent mRNA stability. These findings suggests that inhibition of Mettl-3 plays a role in the development of SLE by promoting CD4^+^T-cell activation and disrupting the balance of effector T-cell differentiation. Therefore, Mettl-3 is essential for stabilizing Foxp3 mRNA through m^6^A modification in supporting Treg differentiation program, highlighting its potential as a therapeutic target for SLE treatment Fig. 2**C,** Table 1.

### CD4^+^T-cells targeting Mettl-3 *(allograft transplantation)*

CD4^+^T-cells activation is major cause of allograft rejection. This study showed the clinical role of Mettl3-expressing CD4^+^T-cells in transplantation setting. The author found that CD4^+^T-cells derived from spleens, draining lymph node and skin allograft abundantly expressing Mettl-3 and higher m^6^A-level. This increases the effector function CD4^+^T-cells making less chance for allograft survival. However, selective inhibition of Mettl-3 by STM2457 (40 µM) impaired CD4^+^T-cells effector program and reduces T-cell proliferation, differentiation into effector T-cells and cell cycle arrest (G0-phase), but augmented cell apoptosis. Furthermore, these impaired T-cell responses were aligned with diminished expression of transcription factors like Ki-67, c-Myc, and T-bet. This suggested the repressed effector function of CD4^+^T-cells by inhibiting Mettl-3, thereby the chances for allograft survival were increased. This indicates the therapeutic role of inhibiting Mettl-3 in CD4^+^T-cells to prevent allograft rejection [134] Fig. 2**D,** Table 1**.**

### CD4^+^T-cells targeting ALKBH5 *(autoimmunity)*

CD4^+^T-cell pathogenicity is one of the major causes of autoimmune disorder was found to be induced by an m^6^A-demethylase ‘ALKBH5′ eraser enzyme. The authors reported that in an animal model of brain inflammation, under experimental autoimmune encephalomyelitis (EAE) condition CD4^+^T-cells is abundantly expressing ALKBH5 and thereby rendering it pathogenic. However, specific deletion of ALKBH5 in CD4^+^T-cells (ALKBH5^f/f^ CD4-CRE mice) confer protection against EAE by reducing pathogenicity. Mechanistically, they showed that inhibition of ALKBH5 increased m^6^A-level of interferon-ƴ and CXCL2 (CXC motif chemokine ligand 2) in CD4^+^T-cells. This causes decreased ‘mRNA stability’ and protein level of IFN-ƴ and CXCL2 resulting in the attenuation of CD4^+^T-cell pathogenicity with diminished neutrophil recruitment in the central nervous system. These findings clearly suggests that ALKBH5 is playing a crucial role in aggravating autoimmunity, and thus targeting ALKBH5 could have therapeutic effect in controlling autoimmune disorder by decreasing the pathogenicity of CD4^+^T-cells (Fig. 2**E**) [135]. However, some other studies showed down-regulated level of ALKBH5, Mettl-14 and YTHDF2 proteins in PBMCs and T-cells of SLE patients [136,137] Table 1.

### CD8^+^T-cells targeting Mettl-3 *(anti-tumor immunity)*

This study revealed the clinical significance of targeting Mettl-3 in increasing the anti-tumor activity of CD8^+^T-cells [138]. The investigators found that the pharmacological inhibition of Mettl-3 by STM3006 (a highly potent 2nd generation Mettl-3 inhibitor than the 1st generation STM2457) has increased the formation of immunogenic double-stranded RNA (dsRNA) and robust cell-intrinsic interferon response within the cancer cells. Experimentally, they validated this dsRNA sensing and interferon activation in a co-culture experiment, in presence of both STM3006 and STM2457, with CD8^+^T-cells and OVA-specific antigen (OT-I) in a range of immunocompetent mice model and later investigated in solid cancers and other hematologic malignancies. Mechanistically, they found that Mettl-3 inhibition globally decreased m^6^A-level leading to (i) increased dsRNA formation, (ii) increased antigen presentation in conjugation with MHC-I and (iii) increased JAK-STAT signalling-mediated activation of interferon associated genes, resulting in increased anti-tumor activity of CD8^+^T-cells. This result suggests that inhibition of Mettl-3 potentiates CD8^+^T-cells killing ability. This research was further summarized and supported by Brewer in 2023 [139]. Moreover, co-therapy targeting Mettl3-inhbitor in combination with anti-PD1 antibody further enhanced the killing ability of CD8^+^T-cells in a mouse model with triple negative breast cancer (TNBC) and prolong survival. These findings revealed the enhanced effectiveness of CD8^+^T-cells in combination with anti-PD1 therapy in boosting anti-tumor immunity (Fig. 2**G,** Table 1). This work was further summarized and supported by Brichkina et al., in 2023 [140]. Similar research showed increased granzymeB^+^IFN-γ^+^CD8^+^T cell-mediated anti-tumor activity in hepatocellular carcinoma (NAFLD-HCC) combining Mettl-3 inhibitor (STM2457) with anti-PD1 therapy, via m^6^A-mediated translation of sterol cleavage-activating protein (SCAP) in the TME [141]. One other study again supported the therapeutic potential of Mettl3-inhibitor (STM2457) in attenuating the immunosuppressive function of tumor-infiltrating myeloid cells (TAMs) in controlling CRC [142]. Conclusively, the combination of Mettl3-inhibitor with anti-PD1 co-therapy showed superior preclinical activity, highlighting its potential in cancer immunotherapy. **STC-15** is a selective inhibitor of Mettl-3 scheduled for phase-I clinical trial (NCT05584111) against advanced malignancies to validate its pharmacological potency and specificity by STORM therapeutics [143] Table 2.

### T_FH-_cells targeting Mettl-3 *(humoral immunity)*

The author has demonstrated the crucial role of Mettl-3 in T-follicular helper cells (T_FH_) differentiation from naïve CD4^+^T-cells, and emphasised its clinical relevance in mediating humoral immunity [144]. T_FH_-cells are one of the specialized subsets of CD4^+^T-helper cells required for B-cell activation in the germinal centre (GC)-follicle to produce specific antibody and memory cells *via* somatic hyper mutation, affinity maturation and by antibody class-switching mechanisms. To understand the selective function of ‘Mettl-3′ in T_FH_ cells the authors has generated CD4^+^T-cell specific conditional knockout mice by selectively depleting ‘Mettl-3′ via crossing Mettl-3^f1/f1^ mice with CD4^cre^ mice (Mettl-3^f1/f1^CD4^cre+^ or Mettl-3^cKO^). Following LCMV infection Mettl3-deficient mice not only showed decreased T_FH_-cell differentiation (CXCR5^+^CD44^+^), but also reduced germinal centre (GC) response/formation as compared to the control wild-type (Mettl-3 ^wt^) littermate mice, confirmed by reduced GC-specific T_FH_-cell differentiation (PD-1^hi^Icos^hi^ Bcl-6^hi^ CXCR5^+^) and B-cell specific (GL-7^+^Fas^+^) markers in the splenic paracortex. This result suggests that ‘Mettl-3′ facilitates T_FH_-cell differentiation and formation of GC-follicle, which is important to produce antibody through CD138^+^ plasma cells. Mechanistically, they showed that Mettl-3 targets 3 -UTR of TCF-7 transcript ‘*encoding TCF-1 protein’* and stabilize its mRNA by recruiting m^6^A-reader (IGF2BPs) proteins [107] confirmed by increased half-life of TCF7-mRNA in wild-type mice (Mettl-3 ^wt^ t_1/2_ = 104 min) as compared to the Mettl-3^mut^ or Mettl-3^fl/fl^ER^T2^-Cre mice (t_1/2_ = 66 min). Moreover, RNA-seq data revealed CISH and SOCS-2 as a potential target of Mettl-3. Furthermore, retroviral overexpression of TCF-7 in Mettl-3^cKO^ mice rescued the T_FH_-differentiation defect. Taken together, these results clearly suggest that (i) TCF-7 is a bona fide target of Mettl-3 and increase its stability by recruiting IGF2BP1-3 reader proteins, (ii) Loss of Mettl-3 impairs T_FH_-cell differentiation and later B-cell activation cascades in the GCs, (iii) Mettl-3 target CISH and SOCS2 in T_FH_-cells, was supported in a similar finding by *Li et al.,* 2017 in CD4^+^T-cells [130] and *Tong et al.,* 2018 in regulatory T-cells [145]. Conclusively, this study revealed the loss of ‘Mettl-3′ in the impairment of T_FH_-differentiation, and hold the potential of utilizing ‘Mettl-3′ as an ‘intracellular checkpoint’ to consider its importance in enhancing B-cell mediated humoral immunity by activating T_FH_-cell differentiation program [144] Fig. 2**H,** Table 1**.**

### Regulatory T-cells targeting Mettl-3 *(inflammatory response)*

**Fig. 3:**
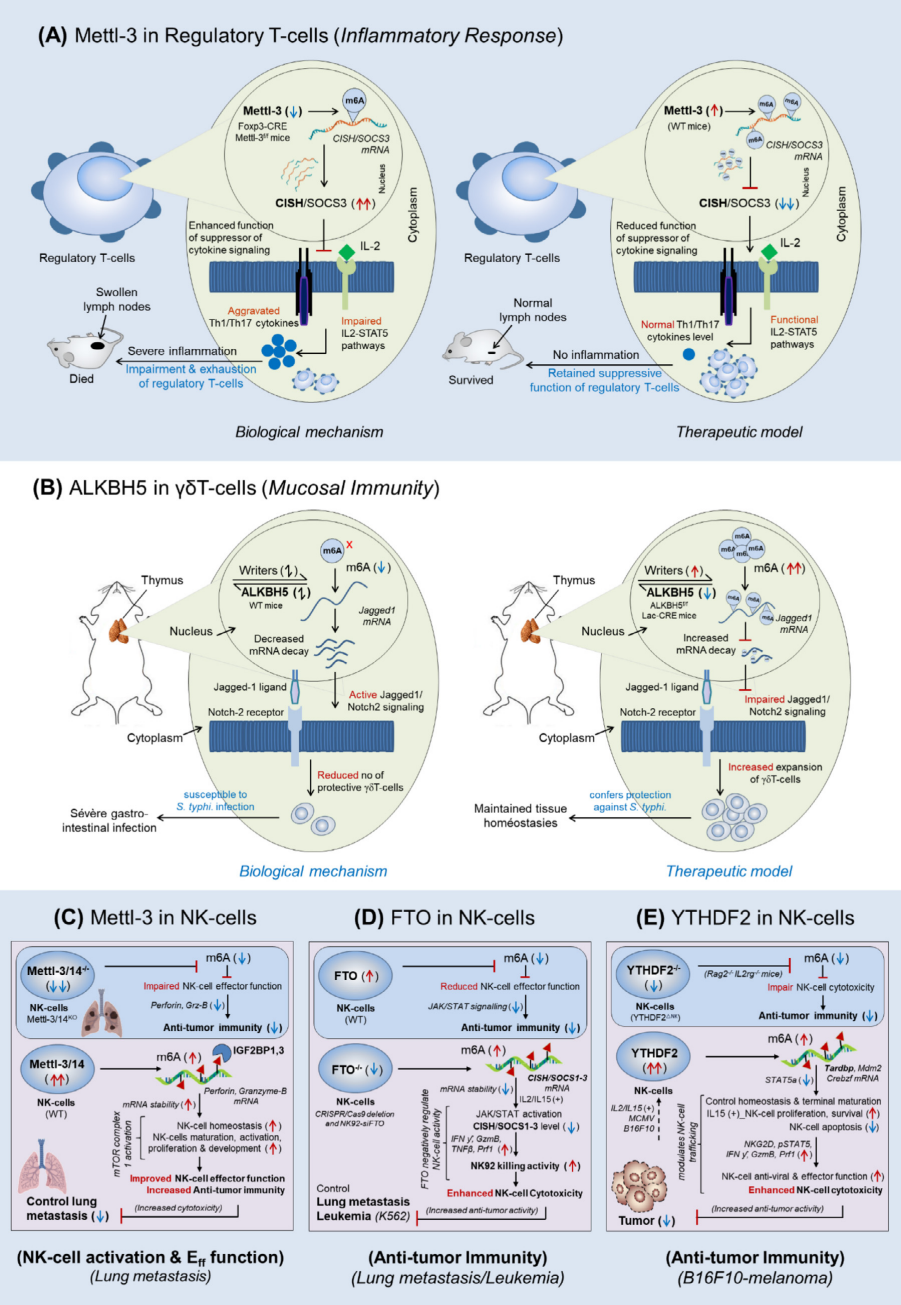
m^6^A-modifiers in regulating the immunological function of T-cell subsets and NK-cells. **A***Mettl-3 in regulatory T-cells (inflammatory response)*: T-regs deficient with Mettl-3 (CD4^+^Foxp3-CRE Mettl-3^f/f^) mice aggravate inflammatory response by releasing (Th1, Th17) cytokines resulting in severe inflammation due to impaired function of regulatory T-cells (*Biological mechanism*). However, wild type T-regs expressing Mettl-3 retain T-reg suppressive function and reduce inflammatory response by inhibiting the master regulator of cytokine signalling CISH and SOCS-3 (*Therapeutic model*). **B***ALKBH5 in γδT-cells (mucosal immunity)*: The up-regulated level of ALKBH5 in γδT-cells prevent its proliferation in peripheral tissues causing susceptibility to *Salmonella typhimurium* infection resulting in severe gastrointestinal infections due to impaired jagged1/notch2 signaling (*Biological mechanism*). However, selective depletion of ALKBH5 in γδT-cells (ALKBH5^f/f^ Lac-CRE mice) enabled m^6^A-mediated degradation of jagged1/notch2 signaling targets resulting in increased abundance of γδT-cells at the peripheral tissues. This provides protection against *S. typhi* infection by increasing mucosal immunity (*Therapeutic model*). **C** Mettl-3/14 in NK-cells *(NK-cell activation and effector function)*: Mettl-3 and Mettl-14 is required for NK-cell activation and to enhance anti-tumor immunity of NK-cells by stabilizing IGF2BP1-mediated effector molecules. **D** FTO in NK-cells *(anti-tumor immunity)*: FTO-inhibition is required to increase the anti-tumor immunity of NK-cells against lung metastasis and leukemia by de-stabilizing the expression of suppressor of cytokine signalling (CISH and SOCS1-3) genes. **E** YTHDF2 in NK-cells *(anti-tumor immunity)*: YTHDF2 is required to increase the anti-tumor immunity of NK-cell against B16F10-tumor by targeting ‘Tardbp’ genes.

This study showed the importance of Mettl-3 in regulating the suppressive function of regulatory T-cells (T-regs) [145]. Building the prior study by *Li et al.,* for the requirement of Mettl-3 in maintaining CD4^+^T-cell homeostasis, and its deficiency in disrupting CD4^+^T-cell proliferation and differentiation [130], with this findings the author anticipated that Mettl3-deficiency might also disrupt regulatory T-cell (CD4^+^FoxP3^+^) function. Therefore, the author observed those mice deficient with Mettl-3 in CD4^+^T-cells (Mettl-3^f/f^ CD4-CRE) for a longer period and found that these mice were normal only up to 3-months but subsequently started developing intestinal inflammations. This clue encouraged them to generate Foxp3-specific conditional knockout mice depleted with Mettl-3 (Mettl-3^f/f^ CD4^+^Foxp3-CRE) to check the selective function of regulatory T-cells. They observed that (Mettl-3^f/f^ Foxp3-CRE) mice have significantly increased the level of inflammatory (Th1 and Th17) cytokines as compared to the wild-type mice, leading to uncontrolled inflammation of the lymph nodes, which exhausted major T-regs population, resulting in the development of severe autoimmune disease, and later become infertile and started dying at the age of 8–9 weeks. This result suggests that regulatory T-cells have lost its suppressive function due to the loss of Mettl-3. Mechanistically, they found that loss of Mettl-3 rescued the up-regulation of SOCS-3 and CISH expression, which in-turn suppressed IL2-STAT5 pathways, resulting in impaired and exhausted T-reg population. Conversely, wild type T-regs suppressed CISH expression which in-turn controlled inflammatory cytokine response and retained T-reg suppressive function. These results clearly suggest that Mettl-3 target CISH and SOCS3 in regulatory T-cells, supported also in a similar finding by *Li et al.,* 2017 in CD4^+^T-cells [130] and *Yao et al.,* 2021 in T_FH_-cells [144]. In conclusion, this study highlighted the crucial role of Mettl-3 in retaining the suppressive function of regulatory T-cells in regulating inflammatory response Fig. 3**A,** Table 1.

Some other studies have also demonstrated the therapeutic potential of Mettl3-inhibition (STM2457 or siMettl-3) in controlling colorectal cancer in models such as HCT116, HT-29, and CT26-induced tumors, primarily by enhancing regulatory T-cell infiltration. Furthermore, combining a Mettl-3 inhibitor with sh-circQSOX1 and anti-CTLA4 presents a promising approach to overcoming T-reg cell-mediated resistance to CRC immunotherapy [146].

### Regulatory T-cells targeting Mettl-14 *(allograft transplantation)*

The author explored the clinical relevance of Mettl14-expressing CD4^+^Foxp3^+^ regulatory T-cells in promoting allograft acceptance using a mouse model of islet transplantation [43]. The author showed that mice with Foxp3-specific deletion of Mettl-14 (Foxp3-Mettl-14^f/+^cKO) exhibited impaired T-reg expansion, as evidenced by reduced expression of IL-10 and TGF-β cytokines in T-regs. This deficiency led to diminished suppressive function of regulatory T-cells, promoting increased CD4^+^ and CD8^+^T-cells infiltration around the allograft resulting in rapid rejection of the transplanted islet allograft. Mechanistically, they identified that the reduced suppressive function of T-regs was due to alteration in SOCS1-3-associated signalling pathways, driven by Mettl14-mediated m^6^A methylation. These findings suggest that Mettl14-mediated m^6^A modification is required to maintain the suppressive function of regulatory T-cells in transplantation and could serve as a key regulatory factor for Treg cell-based therapies in transplant medicine Fig. 2**F,** Table 1.

### Regulatory T-cells targeting YTHDF2 *(anti-tumor immunity: melanoma, CRC)*

This study elucidates the critical role of YTHDF2 inhibition in enhancing the anti-tumor activity of intratumoral regulatory T-cells. The authors demonstrated that selective deletion of YTHDF2 in T-regs (YTHDF2^f/f^Foxp3^Cre^) enhances their suppressive function, leading to increased apoptosis and reduced tumor growth in murine models. Mechanistically, they identified that tumor-derived TNFα induces YTHDF2-mediated degradation of m^6^A-modified transcripts encoding negative regulators of NF-κB signalling, including Nlrc3, Nfkbie, and Traf3. This degradation promotes the survival of functionally impaired T-regs, thereby sustaining an immunosuppressive tumor microenvironment. These findings highlight the therapeutic potential of YTHDF2-inhibition in modulating T-reg populations and improving anti-tumor immunity by reducing the persistence of dysfunctional T-regs, thereby restoring immune balance within the tumor microenvironment [147] Table 1.

### γδT-cells targeting ALKBH5 *(mucosal immunity)*

γδ T-cells are subset of T lymphocytes playing a distinct function in immune surveillance. This study explained the role γδT-cells expressing m^6^A-eraser protein ‘ALKBH5′ (m^6^A-demethylase) in regulating mucosal immunity [148]. The author showed that specific deletion of ALKBH5 in γδT-cells increased proliferation of γδT-cells precursors in thymus & other peripheral tissues and better protected against *Salmonella typhimurium* (*S. typhi.*) infection as compared to the WT-littermates in a mouse model with colonic infection. This suggests ALKBH5 expression prevent γδT-cells expansion and inefficient to protect from external infections*.* Mechanistically, they found that the loss of ALKBH5 increases m^6^A-abundance and methylate Jagged-1 mRNA (jagged canonical Notch ligand 1) leading to increased mRNA decay, resulting in impaired jagged1/notch2-signalling, known to be essential for T-cell lineage commitment. Conclusively, this study revealed the importance of ALKBH5 eraser enzyme in providing mucosal immunity in peripheral tissues, and thus potentiates its therapeutic implications by increasing the surveillance of γδT-cells by silencing intracellular ALKBH5 in γδT-cells [148] Fig. 3**B,** Table 1.

### γδT1 and γδT17-cells targeting Mettl-3 *(γδT-cell differentiation/psoriasis)*

γδT1 and γδT17-cells are the main subsets of γδT-cells, essential for maintaining tissue homeostasis and immunosurveillance. Dysregulation or imbalance between these subsets contributes to skin inflammation, manifesting as psoriasis. This study underscores the critical role of Mettl3-mediated m^6^A modification in γδT-cell differentiation and its significance in the pathogenesis of psoriasis [149]. The authors found that γδT cell-specific deletion of Mettl-3 (Mettl3^f/f^γδT-CD2-CRE) leads to reduced Th-17 cytokine levels, decreased dsRNA formation, consistent with findings by Guirguis et al., 2023, following STM3006 treatment (Fig. 2**G**), and enhanced STAT-1 signalling and IFNγ production through γδT1 cells, suggesting a protective role of γδT1-cells by IFNγ. Mechanistically, they showed that in wild-type (Mettl3^WT^) γδT-cells, Mettl3-mediated m^6^A methylation accelerates the degradation of STAT-1 mRNA, leading to suppressed STAT-1 signalling. This suppression reduces γδT1-differentiation, and thus IFNγ secretion, but promoted γδT17-differentiation and prevented the aberrant formation of endogenous dsRNA. These findings suggest that Mettl-3 plays a critical role in exacerbating psoriasis by driving γδT-cell differentiation toward the γδT-17 phenotype, Table 1.

## B-cells

### B-cells targeting Mettl-14 *(B-cell development & GC-response)*

B-cell development begins with HSCs, MPPs, CLPs followed by the transition from pro-B cells (progenitor B-cells) to large pre-B cells (precursor B-cells), to small pre-B cells, and finally to immature B-cells, which mature into naïve B-cells. The author has highlighted the critical role of Mettl-14 in early B-cell development [150]. Their findings showed that Mettl-14 knockout (Mettl-14^KO^) mice exhibited severe blockade in B-cell development and impaired IL7-induced pro-B cell proliferation as well as the transition from large pre-B cells to small pre-B cells. These defects result in significant disruptions to gene expression programs essential for B-cell development. Mechanistically, they demonstrated that Mettl14-mediated m^6^A modification regulates stages of B-cell development through YTHDF2-dependent pathways and mechanisms independent of YTHDF1 or YTHDF2. Other study also supports the role of YTHDF2 in GC formation [151]. These findings suggest that Mettl14-mediated m^6^A modification plays a critical regulatory role in early B-cell development. However, another study highlighted the role of Mettl-3 in stage-specific development rather than having a direct impact on B-cell development [152].

A similar study further underscores the necessity of Mettl-14 in the germinal center (GC) response, highlighting its role in generating memory B-cells and long-lived antibody-secreting plasma cells [153]. The author demonstrated that Mettl-14 ablation in B-cells (Mettl-14^f/f^CD19-CRE) impairs GC B-cell proliferation and antibody response. Mechanistically, they found that Mettl14-mediated m^6^A modification recruits YTHDF2 to degrade negative immune regulators like Lax1 and Tipe2, thereby upregulating genes essential for GC B-cell selection, cell cycle, and proliferation. Collectively, these findings indicate that Mettl14-mediated m^6^A modification is crucial for B-cell development and germinal center responses Fig. 2**I,** Table 1.

### B-cells targeting Mettl-3 *(DLBCL)*

Diffuse large B-cell lymphoma (DLBCL) is the most common subtype of lymphoma, accounting for 30–40 % of all adult non-Hodgkin lymphoma cases. Despite the excellent response of Kymriah® (*Tisagenlecleucel*) and Yescarta® (*Axicabtagene Ciloleucel*), anti-CD19 CAR T-cell therapy, to treat different subtypes of lymphoma (ALL, DLBCL, HNL). A significant number of patients experience relapse within 6–7 months of treatment [154]. The study, by Cheng et al, 2020 highlights the pivotal role of Mettl-3 inhibition in reducing DLBCL proliferation by targeting pigment epithelium-derived factor (PEDF). These findings suggest that combining a Mettl-3 inhibitor with CAR T-cell therapy could enhance its therapeutic efficacy in controlling DLBCL [155] Table 1. Furthermore, Mettl-3 or YTHDF2 knockdown in Farage/R cells (human B lymphocyte cell line) has been shown in reducing Rituximab resistance by upregulating C1qA expression [156]. YTHDF2 serves as an oncogene in DLBCL, and its knockdown has been shown in reducing DLBCL proliferation by modulating ACER2-mediated ceramide metabolism [157].

## Natural killer cells (NK-cells)

### NK-cells targeting Mettl-3 *(NK-cell activation and effector function/lung metastasis)*

The author highlights the critical role of Mettl-3 in the development and effector functions of NK cells [158]. Their findings demonstrate that Mettl-3 and Mettl-14-mediated m6A methylation activates NK-cells when supplemented with IL-2, IL-12, and IL-15 in NK cell lines, while this activation is suppressed in tumor (hypoxic) microenvironments. The double knockout of Mettl-3 and Mettl-14 significantly disrupts NK-cell homeostasis, maturation (marked by CD27 and CD11b), and antitumor immunity (indicated by CD69, FasL, Perforin, and Granzyme-B). Mechanistically, RNA immunoprecipitation sequencing (RNA IP-seq) revealed that m6A methylation stabilizes the mRNA of perforin and granzyme-B by recruiting IGF2BP1 and IGF2BP3 reader proteins. Additionally, the inhibition of mTOR complex 1 activation prevented the elevation of m6A methylation levels during NK-cell activation, which could be restored through S-adenosylmethionine supplementation. These findings underscore the requirement of Mettl-3 for enhancing the anti-tumor activity of NK-cells, particularly in controlling lung metastasis, Fig. 3**C,** Table 1.

### NK-cells targeting Mettl-3 *(anti-tumor immunity/lymphoma)*

The investigator found the importance of m^6^A writer protein ‘Mettl-3′ in increasing NK cell-mediated anti-tumor immunity [159]. The author observed that tumor-infiltrating NK-cells have decreased level of Mettl-3 causes altered homeostasis, reduced infiltration and impaired effector function in the tumor microenvironment (TME) resulting in accelerated tumor growth and decreased mice survival. Mechanistically, Mettl3-mediated m^6^A methylation helps NK-cells respond to IL-15 by supporting signalling pathways downstream of IL-15R in the TME. The SHP-2 gene (Src homology-2 protein tyrosine phosphatase), modified by m^6^A, shows reduced protein expression in Mettl3-deficient NK-cells, leading to lower SHP-2 activity. This decreases NK-cell responsiveness to IL-15 and weakens AKT and MAPK pathway activation. As a result, Mettl3-deficient NK-cells have reduced ability to kill YAC-1 lymphoma cells in vitro compared to normal NK-cells. These findings highlight the importance of Mettl-3 in maintaining NK-cell homeostasis and tumor immunosurveillance, suggesting its potential as a therapeutic target for enhancing NK cell-mediated anti-tumor immunity (Table 1). One another study highlights the role of m^6^A-writer enzymes WTAP’s in NKTCL progression and drug resistance. WTAP is overexpressed, stabilizes DUSP6 mRNA via m^6^A-methylation, and promotes proliferation and cisplatin resistance. Targeting the WTAP-DUSP6 axis may enhance cisplatin sensitivity and improve NK cell-based immunotherapies for NKTCL treatment [160]. A similar study further supports the role of Mettl-3 in NKTCL progression and cisplatin resistance. Mettl-3 enhances m^6^A-modification and stabilizes SND1 mRNA, increasing SND1 expression. This promotes tumor cell survival and drug resistance, suggesting that targeting the Mettl3-SND1 axis could improve NKTCL treatment outcomes [161] Table 1.

### NK-cells targeting FTO *(anti-tumor activity/lung metastasis & leukemia)*

The study demonstrates the therapeutic potential of inhibiting FTO, an m6A demethylase enzyme, in enhancing the cytotoxic activity of NK-cells. Wild-type NK cells expressing FTO, derived from the spleen and bone marrow of mice, were associated with a higher number of metastatic nodules in the lungs, increased tumor burden, and reduced survival compared to CRISPR/Cas9-mediated FTO knockout (FTO^-/-^) mice under B16F10 melanoma conditions. Additionally, splenic NK-cells from wild-type mice exhibited reduced levels of interferon-γ (IFN-γ), leading to diminished cytotoxic activity against the EL4 T-cell lymphoma cell line, relative to FTO-deficient NK cells. In parallel, NK92 cells with siRNA-mediated FTO knockdown (siFTO) displayed enhanced killing activity against the K562 leukemia cell line compared to control siRNA-treated (siCTRL) NK92 cells. Mechanistically, siFTONK92-cells demonstrated upregulation of the JAK/STAT signalling pathway and its downstream targets, including granzyme B, IFN-γ, and TNF-α, further supporting enhanced cytotoxic function. Notably, siFTONK92-cells showed elevated expression of SOCS family genes (**CISH**, SOCS1, SOCS2, and SOCS3). m6A-methylated RNA immunoprecipitation (MeRIP) analysis revealed that SOCS1 and SOCS3 are de-stabilized by m^6^A-mediated modifications, as indicated by their decreased levels in siFTONK92-cells compared to siCTRL cells. Collectively, these findings revealed that FTO is negatively regulating NK-cell function. Thus, FTO inhibition emerges as a promising intracellular checkpoint strategy to enhance NK cell-mediated anti-tumor immunity, potentially improving the control of lung metastasis and leukemia [162] Fig. 3**D,** Table 1.

### NK-cells targeting YTHDF2 *(anti-tumor immunity/B16F10-tumor)*

This study illustrated the crucial role of m^6^A reader protein ‘YTHDF2′ in NK cell-mediated anti-tumor immunity. The author investigated that YTHDF2 is upregulated in NK-cells upon activation by cytokines, B16F10-mediated tumors or by cytomegalovirus (MCMV) infection. Conversely, YTHDF2-deficiency in NK-cells showed impaired anti-tumor and anti-viral activity in vivo. They further investigated that YTHDF2 is required for the maintenance of NK-cell homeostasis, terminal maturation, modulating NK-cell trafficking, regulating eomes expression (eomesodermin: T-box transcription factor like T-bet) and promoting NK-cell effector function. In addition, they identified YTHDF2 is required for IL15-mediated NK-cell survival and proliferation by forming a STAT5-YTHDF2 positive feedback loop. In contrast, YTHDF2-deficiency showed decreased expression of IFN-γ, granzyme B, and perforin leading to increased tumor metastases and reduced NK-cell infiltration in tumor mouse model. Mechanistically, they identified Tardbp (TAR DNA-binding protein) as a target of YTHDF2 involved in NK-cell proliferation or survival. These findings stress the importance of YTHDF2 in regulating NK-cell anti-tumor immunity and suggest its potential as a therapeutic target for enhancing NK cell-mediated cancer immunotherapy [163] (Fig. 3**E**). One another study also supports the importance of YTHDF2 in enhancing NK-cell cytotoxic activity against colorectal cancer via SMAD4 through increasing the expression of NKG2D and granzyme-B levels [164] Table 1.

## Dendritic cells (DCs)

### Dendritic cells targeting Mettl-3 *(DC-activation)*

**Fig. 4:**
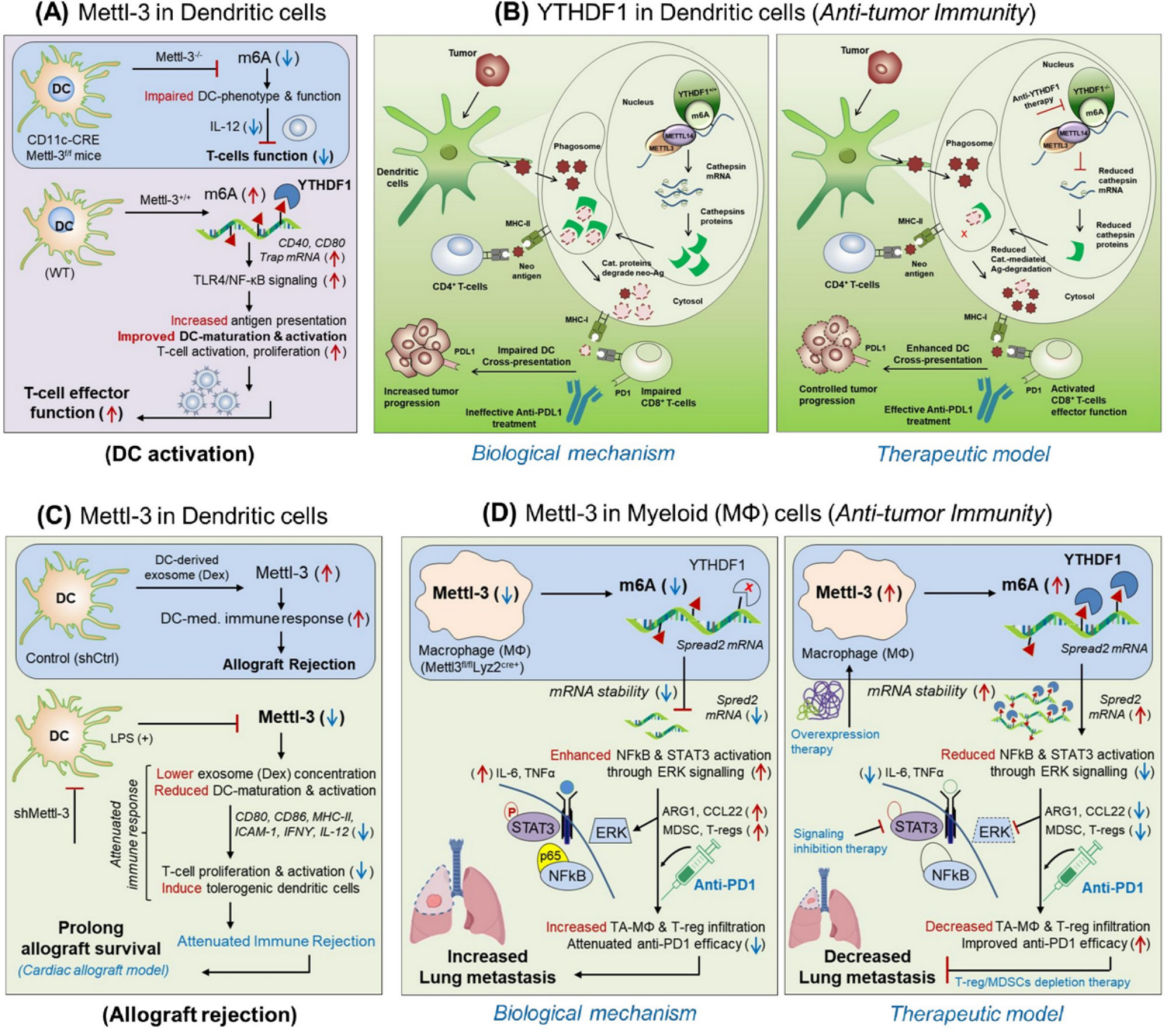
m^6^A-modifiers in regulating the immunological function of immune cell subsets. **A***Mettl-3 in dendritic cells (DC-activation)*: Mettl-3 promotes dendritic cell maturation and activation by increasing antigen presentation capability and co-stimulatory molecule expression, evidenced by upregulated expression of CD40, CD86, IL-12 (cytokines), and by activated TLR4/NF-κB signaling by recruiting YTHDF1 reader proteins, resulting in DC-mediated enhancement of T-cell activation and effector function. **B***YTHDF1 in dendritic cells (anti-tumor immunity)*: *Biological mechanism*: YTHDF1 recognizes m^6^A-marked cathepsin transcripts in the DCs and increases its expression, which translocate into the phagosomal compartment and degrades neo-antigens. This process limits antigen recognition and cross-presentation resulting in impaired DC-mediated CD8^+^T-cell effector function. *Therapeutic model*: proposed anti-YTHDF1 therapy: targeting YTHDF1 in DCs would reduce cathepsin level and thus unable to degrade neo-antigens leading to better antigen recognition and cross-presentation to CD8^+^T-cells resulting in increased DC-mediated anti-tumor activity and thus improved efficacy of anti-PD1 therapy. **C***Mettl-3 in dendritic cells (allograft rejection)*: Mettl-3 is required for DC-activation and thus increases T-cell effector function resulting in decreased allograft survival. However, Metttl-3 inhibition in DC (shMettl-3) prevent cardiac transplant by reducing DC-mediated antigen presentation and T-cell activation evidenced by decreased expression of co-stimulatory molecules and cytokines. **D***Mettl-3 in macrophage cells (anti-tumor immunity/lung metastasis)*: Mettl-3 ablation promotes tumor progression by enhancing M1/M2-like tumor-associated macrophage and regulatory T-cell infiltration in the TME. This occurs via YTHDF1-mediated suppression of Spread2 mRNA, driven by NFkB-STAT3 activation through the ERK pathway. Additionally, it reduces anti-PD1 efficacy in Mettl3-deficient mice (*Biological mechanism*).: Mettl-3 overexpression control tumor progression by improving anti-PD1 efficacy and inhibiting the recruitment of M1/M2-like tumor-associated macrophages and regulatory T-cells in the TME occur via YTHDF1-mediated increased translation of Spread2 and NFkB-STAT3 inactivation (*Therapeutic model*).

This study highlights the significant role of Mettl-3 in dendritic cell (DCs) activation. To understand the specific role of Mettl-3 in dendritic cells a conditional knockout (Mettl-3^cKO^) mice were generated by crossing Mettl-3^f/f^ and CD11c-Cre mice. Mettl3-deficient DC showed impaired antigen presentation, maturation and activation confirmed diminished expression of co-stimulatory molecules (CD40, CD80) and pro-inflammatory cytokines (IL-6, IL-12p70, TNFα), resulting in attenuated inflammatory response and reduced DC-mediated T-cell activation. Mechanistically, they identified that Mettl3-mediated m^6^A methylation enhanced the expression of CD40, CD80 and the TLR4 signalling adaptor Tirap through YTHDF1-mediated mRNA translation (Fig. 1**A**). This process strengthens TLR4/NF-κB signalling and cytokine production, which are essential for T-cell activation. This result suggests that (i) Mettl-3 is playing a crucial role in DC-activation and (ii) Mettl-3 promote DC function by targeting TLR4/NF-κB signalling pathway through the recruitment of YTHDF1 reader proteins. In conclusion, Mettl3-m^6^A axis in dendritic cells represents a potential therapeutic target in regulating pathological inflammation and enhancing anti-tumor immunity. This axis offers a promising molecular target for advancing cancer immunotherapy strategies [165] Fig. 4**A**, Table 1**.**

### Dendritic cells targeting YTHDF1 *(anti-tumor immunity)*

Insufficient anti-tumor immunity is the major drawback in cancer immunotherapy. This study demonstrates the significant role of dendritic cells expressing m^6^A-reader protein ‘YTHDF1′ in regulating anti-tumor immunity [166]. The study showed that dendritic cells deficient with YTHDF1 (YTHDF1^cKO^) enhanced antigen-recognition and cross-presentation ability of DCs in-vivo, resulting in elevated antigen specific CD8^+^T-cell response as compared to the control wild type (YTHDF1^WT^) mice. Moreover, YTHDF1^cKO^ mice showed increased efficacy of anti-PD1 checkpoint blockade therapy. Mechanistically, they found that the wild type mice, m^6^A-methylation recruited YTHDF1 reader proteins at the lysosomal-cathepsins mRNA axis, resulting in increased mRNA-stability, and thus increased abundance of cathepsin proteins in the phagosomal compartments of the DCs. This caused severe degradation of the neo-antigens and thus limiting the antigen availability to the DCs for antigen-recognition and further cross-presentation to CD8^+^T-cells in the cytosol (Fig. 4**B**, *biological mechanism*). This result suggests that YTHDF1 is playing a crucial role in suppressing anti-tumor immunity. Therefore, silencing intracellular YTHDF1 in DCs would be a potential approach to enhance anti-tumor immunity. Collectively, this discovery improves two immunotherapeutic procedures by co-targeting (i) anti-YTHDF1 therapy: where, YTHDF1-deficiency protect ‘antigen-degradation’ and allows efficient recognition and presentation by DCs, in-turn, further increases the abundance of effector CD8^+^T-cells by cross-presentation mechanism and (ii) anti-PD1/PDL1 co-therapy: would further potentiates the efficacy of anti-PD1 immunotherapeutics by enhancing the effector function of CD8^+^T-cells (Fig. 4**B**, *therapeutic model*), Table 1. Similar research was summarized and supported by Kim et al., in 2019 [167]. One more study further supports the loss of YTHDF1 in enhancing DC-mediated anti-tumor immunity. Specifically, inhibition of YTHDF1 (shYTHDF1) in gastric cancer cell lines (AGS, BGC823, MKN74, and YTH16) increased the infiltration of mature DCs with elevated expression of MHC-II and IL-12 compared to siControl. This, in turn, promoted the infiltration of CD4^+^T-cells and CD8^+^T-cells, accompanied by an increased expression of IFNγ [168].

### Dendritic cells targeting Mettl-3 *(cardiac transplant)*

Activated dendritic cell is the major cause of allograft rejection. This study showed the clinical use of ‘DC-derived exosomes’ in cardiac allograft survival in a mouse model with heart transplant [169]. Exosomes are extracellular vesicle containing ‘nucleoproteins’ carrying therapeutic values, however, sometimes it also affects the behaviour of adjacent cells/tissues/organs upon secretion. The author has explored the key role of m^6^A-writer enzyme ‘Mettl-3′ in secreting DC-derived exosomes during organ transplantation. The author found that, Mettl3-silenced mature DCs (shMettl3-mDCs) limits exosome secretion, mirroring tolerogenic DC-behaviour instead of immunogenic property and thus inhibits the activation of immune cascade. This hypothesis was also supported by Wang et al., 2019; wherein, Mettl-3 was required for DC-maturation and T-cell activation [165,170]. In a cardiac transplant operation, six days prior to the heart transplantation, the injection of in vitro co-cultured ‘exosomes’ (derived from Mettl-3 silenced mature DCs ‘shMettl3-mDex’ isolated from donor BALB/c mice bone marrow, and splenic ‘T-lymphocytes’ from recipient mice) into C57BL/6 recipient mice has limited exosome transfer, confirmed by lower expression of ICAM-1, MHC-I and MHC-II specific exosome markers, resulting in decreased T cell-mediated immune aggravations and thereby prevents allograft rejection as compared to the control (shCtrl-mDex) injected cells. This result suggests that Mettl-3 is playing a crucial role in blocking exosome transfer and preventing DC-mediated immune cells activations (Fig. 4**C,** Table 1). Conclusively, this study reveals the immunotherapeutic value of DC-derived exosomes in prolonging heart transplant survival by targeting intracellular Mettl-3 in DCs. A previous study also supports the similar hypothesis by *Wu et al., 2020* [44].

## Macrophages (MΦ)

### Macrophage targeting Mettl-3 *(anti-inflammatory response)*

The author has explained the role of Mettl-3 in priming pro-inflammatory reactions and enlightened the molecular mechanism to control inflammatory diseases [171]. They showed that M1-polarization (pro-inflammatory), M2 (anti-inflammatory) and M−reg (immune-suppressive) polarization is essential for maintaining cellular homeostasis. They found that Mettl-3 is highly expressed in murine bone marrow derived macrophage (BMDMs) and induces M1-polarization. However, knockdown Mettl-3 (siMettl-3) in macrophage, resulting in reduced M1-polarization but enhanced M2-polarization compared to the control (scramble siRNA duplex) transfections. Conversely, overexpression of Mettl-3 (pcDNA3.1 expressing Mettl-3) facilitated M1-polarization but attenuated M2-polarization. This result suggests that Mettl-3 positively regulates M1-polarization. Mechanistically, they justified that Mettl-3 (via RNA-immunoprecipitation and in-vitro m^6^A-methylation assay) directly target STAT-1 mRNA and methylate by adding m^6^A-methylation mark, resulting in increased mRNA stability and thus upregulated the expression of STAT-1 ‘a master transcription factor in controlling M1-macrophage polarization’ protein. These results clearly suggest that Mettl-3 derives M1-polarization/pro-inflammatory responses by increasing the expression of STAT-1 transcription factor. Conclusively, this study reveals ‘Mettl-3′ could be a potential anti-inflammatory target Table 1. Another study also reported the upregulation of Mettl-3 in rheumatoid arthritis (RA) patients. Mettl-3 regulates inflammation in macrophages through NF-κB, reducing LPS-induced inflammation. These findings highlight Mettl-3 as a potential biomarker and therapeutic target for RA [172] Table 1.

### Macrophage targeting Mettl-3 *(anti-tumor immunity)*

This study highlights the critical role of Mettl-3 in regulating macrophage-mediated anti-tumor immunity. Deletion of Mettl-3 in macrophages (Mettl-3^fl/fl^Lyz2^cre^) significantly increased lung metastasis in vivo. Mettl3-deficient mice exhibited enhanced infiltration of both M1-like (anti-tumor) and M2-like (pro-tumor) tumor-associated macrophages, along with an increased regulatory T-cell population in the tumor microenvironment (TME). Mechanistically, Mettl-3 loss disrupted m^6^A-dependent translation of Spred2 via the YTHDF1 m^6^A-reader protein, leading to hyperactivation of NF-κB and STAT3 signalling through the ERK pathway, promoting tumor progression and metastasis. Additionally, Mettl-3 deficiency impaired responsiveness to PD-1 checkpoint blockade therapy by polarizing M1/M2 macrophages, recruiting immunosuppressive cells in the TME, and reducing the effector function of CD4^+^CD8^+^IFNγ T-cells. Overexpression of Mettl-3 restored Spred2 expression through YTHDF1-dependent translation, identifying Spred2 as a key downstream target of Mettl-3. These findings establish Mettl-3 as essential for enhancing anti-tumor immunity and suggest it as a potential therapeutic target to improve immune checkpoint blockade efficacy Fig. 4**D**, Table 1 [173].

### Macrophage targeting FTO *(MΦ-activation in type-2 diabetes)*

This study shows that FTO-inhibition suppresses M1 and M2 macrophage polarization by downregulating NF-κB signalling and reducing STAT1 and PPAR-γ expression. FTO knockdown destabilizes these mRNAs via YTHDF2, impairing macrophage activation, highlighting FTO as a potential target for immune regulation [174]. One another study further highlights the role of macrophage-expressed FTO and miR-495 in type 2 diabetes (T2D). miR-495 inhibits FTO, promoting pro-inflammatory M1 macrophage polarization, exacerbating insulin resistance and adipose inflammation. Targeting the miR-495/FTO axis by inhibiting miR-495 or enhancing FTO may offer a therapeutic strategy to improve insulin sensitivity in T2D [56] Table 1.

### Macrophage targeting YTHDF2 *(inflammatory response)*

This study showed the role of m^6^A-reader protein ‘YTHDF2′ in controlling inflammatory responses [175]. They found that the siRNA-depletion of YTHDF2 in RAW 264.7 cells (derived from mouse monocyte macrophage) increases the expression of pro-inflammatory cytokines IL-6, TNF-α, IL-1β and IL-12 upon LPS stimulation, suggesting the protective role of YTHDF2 in inflammatory reactions. Mechanistically, they elucidated that YTHDF2-deficiency stabilizes mRNA of MAP2K4 and MAP4K4 transcript by lowering its mRNA decay and thereby increasing the activity of MAPK and NF-κB signalling pathways, confirmed by phosphorylation of p65, p38 and ERK1/2 intermediates. Moreover, specific inhibition of NFκB-MAPK signalling by selective NF-κB (BAY 11–7082), p38 (SB203580) and ERK (U0126) inhibitors significantly decreases the expression of IL-6 and TNF-α cytokines under YTHDF2-deficient state, indicating NF-κB and MAPK signalling pathway has a crucial role in upregulating inflammatory processes. Collectively, this study suggests that YTHDF2 is required to control inflammatory aggravations by de-stabilizing (increasing) mRNA decay of MAP2K4 and MAP4K4 transcript leading to the downregulation of the NFκB-MAPK activity. This study further implicates the therapeutic ability of YTHDF2 in controlling inflammatory reactions and thus emphasizing ‘YTHDF2′ as a potential anti-inflammatory target in LPS-stimulated RAW 264.7 cells Table 1.

### Macrophage targeting IGF2BP2 *(inflammatory disease/colitis)*

In this study the role of ‘IGF2BP2′ has been highlighted in controlling inflammatory diseases by promoting the transition from proinflammatory (M1) to pro-healing (M2) macrophages. It has been shown that IGF2BP2-deletion in macrophages enhanced the M1 phenotype, exacerbating dextran sulfate sodium-induced colitis, while impairing IL-4-induced M2 activation and reducing cockroach extract-induced pulmonary allergic inflammation. Mechanistically, IGF2BP2 facilitates M1-to-M2 transition via m^6^A-dependent targeting of TSC1 and involves the STAT6-HMGA2-IGF2BP2-PPARγ axis. These findings underscore the requirement of IGF2BP2′s in macrophage activation and its potential as a therapeutic target to control inflammatory diseases [176] Table 1.

However, one another study painted the role of ‘YTHDF2′ an m6A-reader protein in tumor immune evasion. They showed that the loss of YTHDF2 inhibited tumor growth in MC38 and B16-ova-induced tumor models and prolonged survival in immunocompetent models. Mechanistically, YTHDF2-deficiency enhanced macrophage recruitment via CX3CL1, improved CD8^+^T-cell mitochondrial respiration by disrupting tumor glycolysis, and promoted inflammatory macrophage polarization and antigen presentation in the presence of IFN-γ. This allowed IFN-γ to induce autophagic degradation of YTHDF2, sensitizing tumor cells to CD8^+^T-cell cytotoxicity. Furthermore, a small molecule (DF-A7) induced YTHDF2 degradation showed potent antitumor effects, enhanced by anti-PD-L1 or anti-PD-1 therapy, suggesting its potential for improving cancer immunotherapy [177] Table 3.

## Myeloid cells

### Tumor-infiltrating myeloid cells (TIMs) targeting Mettl-3 *(immune escape/CRC)*

This study demonstrated that Mettl-3 expression enhances the immunosuppressive capabilities of tumor-infiltrating myeloid cells (TAMs, TANs, MDSCs, and DC-regs) and facilitates immune evasion in a mouse model of colorectal cancer. Mechanistically, the researchers found that excessive lactate accumulation in TIMs upregulates Mettl-3 levels through H3K18 lactylation, thereby increasing m^6^A-modification of JAK1 mRNA. This modification recruits YTHDF1-mediated translation via STAT3 phosphorylation, leading to heightened immunosuppressive activity in TAMs. Importantly, inhibiting Mettl-3 (Mettl3^f1/f1^LysM-cre/STM2457) attenuated tumor growth and prolonged survival in mice. These findings underscore Mettl-3 inhibition as a promising strategy to diminish TAM-mediated immunosuppression and suggest therapeutic potential in TAM reprogramming [142] Table 1, Table 3.

### Tumor-associated macrophage (TAMs) targeting Mettl-14 *(anti-tumor immunity/CRC)*

This study elucidates the essential role of Mettl-14 in tumor-associated macrophages (TAMs) and its significance in promoting anti-tumor immunity in colorectal cancer. The authors demonstrate that Mettl-14 is crucial for facilitating the infiltration and functionality of CD8^+^T-cells within the tumor microenvironment. Specifically, the loss of Mettl-14 in TAMs leads to a marked reduction in tumor-infiltrating CD8^+^T-cells, suggesting its pivotal role in sustaining an effective anti-tumor immune response. These findings underscore the importance of Mettl-14 in regulating TAM-mediated immune surveillance and suggest that targeting RNA m^6^A-modifications in macrophages could be a potential strategy to enhance CD8^+^T-cell-mediated tumor immunity [178] Table 1.

### Tumor-associated macrophage (TAMs) targeting YTHDF2 *(anti-tumor immunity melanoma, CRC)*

This study highlights the crucial role of YTHDF2-inhibition in increasing the anti-tumor immunity of tumor-associated macrophage (TAMs). The research reveals that elevated YTHDF2 levels in TAMs lead to increased degradation of STAT-1 mRNA via m^6^A modification, subsequently reducing IFNγ signalling and impairing CD8^+^T-cell cytotoxicity. Conversely, YTHDF2 deficiency in TAMs (YTHDF2^f/f^Lyz^Cre^) enhances antigen presentation capabilities, thereby boosting CD8^+^T-cell-mediated anti-tumor immune responses. These findings underscore the potential of targeting YTHDF2 as an immunotherapeutic strategy to enhance TAM-driven anti-tumor immunity by promoting CD8^+^T-cell effector functions against melanoma and CRC [179] Table 1.

### MDSCs targeting YTHDF2 *(anti-tumor immunity/lung metastasis)*

This study demonstrated that local tumor irradiation induces the expression of YTHDF2 and promotes the expansion of myeloid-derived suppressor cells (MDSCs) in both murine and human models. The researchers observed that combining ionizing radiation (IR) with YTHDF2-knockout (Lyz^cre^YTHDF2^f1/f1^) resulted in reduced tumor volume, decreased lung metastasis, enhanced CD8^+^ T-cell proliferation, and thus improved survival in MC38, B16-OVA, and LLC-induced tumor models. Additionally, pharmacological inhibition of YTHDF2 (DC-Y13) combined with anti-PD1 therapy followed by radiation further decreased tumor size and enhanced the effectiveness of checkpoint blockade therapy (NCT02608385, NCT03223155). Mechanistically, the study revealed that IR-induced YTHDF2 expression directly binds to and degrades transcripts encoding negative regulators of NF-κB signalling (Tnfaip8l2, **SOCS3**, Smpdl3b, Metrnl, and Adrb2), leading to activation of NF-κB signalling, thereby establishing an IR-YTHDF2-NF-κB circuit. These findings suggest that loss of YTHDF2 in myeloid cells enhances antitumor immunity and overcomes tumor radio resistance by altering MDSC differentiation, inhibiting MDSC infiltration, and suppressing their immunosuppressive functions [180] Table 1, Table 3. Mettl-3 knockdown in CD33^+^MDSCs has been also shown in decreasing MDSCs density in cervical cancer [181].

Another study also corroborates the proteolytic degradation of YTHDF2 through ubiquitination by keratin type I cytoskeletal 17 (KRT17) and thereby inhibiting colorectal cancer progression by increasing cytotoxic T-cell infiltration and anti-PD1 efficacy by targeting CXCL10 [182] Table 3.

## Factors affecting mRNA m^6^A-modification

The major factors affecting mRNA m^6^A-modification are (i) enzymatic modifiers (writers, erasers, readers) [58,183], (ii) physiological conditions (cell type, developmental stage and diseased condition) [184], (iii) RNA structure and sequence (consensus motif and secondary structure) [61,67], (iv) cellular content (transcription rate and cellular localization) [61], (v) external factors (stressed condition and nutrients availability) [185,186], and more importantly (vi) microRNA-mediated regulation (via feed-back regulatory mechanism) [183,187]. Of note, some microRNA can target m^6^A-associated enzymes, creating a feedback loop in the regulation of m^6^A-modifications in turn m^6^A-modification can also regulate microRNA processing by site specific modifications [188]. Additionally, histone H3 trimethylation at lysine 36 (H3K36me3) has been shown to regulate Mettl14-mediated m^6^A modifications, highlighting a crosstalk between H3K36me3 and RNA methylation in gene regulation [29] (Fig. 1**D**). Such regulatory feed-back loop is interesting to understand the overall gene regulation, diseased conditions, and therapeutic approach [189,190].

### microRNA regulating m^6^A-modifiers (*a feed-back mechanism*)

miR-145 modulate YTHDF2 by binding to 3‘-UTR of YTHDF2 mRNA in HCC confirmed by decreased miR145-mediated luciferase activity with 3‘-UTR of YTHDF2 mRNA. Furthermore, overexpression of miR-145 showed dose-dependent downregulation of YTHDF2 with increased m^6^A-level in dot-blot assays [191]. miR-376c target YTHDF1 in NSCLC was confirmed by dual luciferase reporter assay with miR-376c in downregulating YTHDF1 expression and Wnt/β-catenin pathway in endothelial cells [192]. miR-495 target m^6^A-demethylase ‘FTO’ in macrophage-mediated insulin resistance in type 2 diabetes (T2D) [56]. miR1306-5p modulates Mettl-14 mediated m^6^A methylation to suppress acute myeloid leukemia [193].

### m^6^A-modifiers regulating microRNA

m^6^A-methylation facilitates the initiation of microRNA biogenesis [27]. m^6^A-methylation regulate miRNA25-3P maturation in pancreatic cancer [194]. Mettl-3 regulate miR-186-5p and promote docetaxel resistance in triple negative breast cancer [195]. Mettl-3 promotes pri-microRNA-221-3p maturation by accelerating adriamycin resistance in MCF-7 breast cancer cells [196]. Mettl-3 regulate miR-1246/SPRED2/MAPK signalling in promoting colorectal cancer [197]. Mettl-14 regulate miR-126 through miRNA-processing in hepatocellular carcinoma (HCC) [198]. Mettl-14 regulate miR-375 in spinal cord injury [199]. An m^6^A-eraser enzyme ‘FTO’ regulates cell migration and invasion in breast cancer through the miR-181b-3p/ARL5B signalling pathway [200]. ALKBH5 downregulate pre-miR181b-1 by targeting YTHDF2-mediated degradation and oncogenic YAP expression, and YTHDF1-mediated translation in osteosarcoma [50]. hnRNPA2/B1 regulates primary microRNA processing [103]. All these investigations clearly suggest the feedback regulatory mechanism of m^6^A-modification in regulating microRNA and vice versa.

## Anti-PD1 targeted therapy Vs m^6^A-modifiers

A significant clinical success has been achieved in the treatment of various cancers through immunotherapy employing programmed cell death-1 (PD-1) checkpoint blockade. For example, OPDIVO® (Nivolumab) and KEYTRUDA® (Pembrolizumab) have revolutionized cancer immunotherapy by reactivating T-cells to attack tumors. However, the limited therapeutic response has been observed in most of the patients receiving these antibodies remains a critical challenge, necessitating a deeper understanding of the molecular mechanisms that restrict the efficacy of immunotherapy. Emerging evidence suggests that targeting m^6^A-modification machinery in conjunction with anti-PD-1 therapy may exert a synergistic effect in enhancing the therapeutic potential. Kumar et al. (2021) has comprehensively elucidated the role of m^6^A-modifiers in improving the outcomes of immune checkpoint-based therapies [201]. In this section, we reviewed recent advancement in personalized therapy co-targeting m^6^A-modification enzymes alongside anti-PD-1 therapy to further augment the functional capacity of immune cells.

### Adverse effect of high-dose antibody-based targeted therapy

Higher antibody doses are often required after initial treatment due to the resistance developed by the patient. However, escalating the dose increases the risk of immune-related adverse events, reflecting excessive immune activation and impaired self-tolerance. Common side effects include fatigue, rash, pruritus, and diarrhoea, while severe toxicities may affect organs such as the lungs (pneumonitis), liver (hepatitis), colon (colitis), endocrine glands (thyroiditis, adrenal insufficiency, hypophysis), and, less frequently, the heart (myocarditis) [202]. Infusion reactions, though rare, can present as fever, chills, hypotension, or dyspnea. In rare instances, severe immune dysregulation may cause life-threatening complications like cytokine release syndrome (CRS) or multi-organ failure. Thus, while high-dose anti-PD-1 therapy may improve antitumor efficacy, it also poses significant safety risks that must be carefully balanced against clinical benefit [203].

### Co-targeting m^6^A-modifiers in improving anti-PD-1 efficacy

These studies have demonstrated the enhanced therapeutic efficacy of anti-PD-1 antibodies when combined with m^6^A modifiers. For example, (i) CD8^+^T-cells targeting ‘Mettl3′ using STM2457-inhibitor has shown remarkable increase in anti-tumor activity when given in combination with anti-PD1 therapy [138,140]. (ii) Mettl-3 inhibitor showed enhanced activity of CD8^+^T in controlling non-alcoholic fatty liver disease (NAFLD) a type of hepatocellular carcinoma, when administered in combination with anti-PD1 [141]. (iii) Mettl-3 and Mettl-14 inhibition by CRISPR-Cas (sgRNA) in tumor (CT26, B16) cell line showed increased infiltration of cytotoxic CD8^+^T-cells in the TME and elevated IFN-γ, CXCL9, and CXCL10 levels through enhanced expression of STAT1 and IRF1, mediated by YTHDF2-dependent mRNA stabilization. Moreover, combining anti-PD1 further improved its efficacy in controlling pMMR-MSI-L colorectal carcinoma (CRC) and melanoma by boosting cytotoxic immune responses and IFN-γ signalling [204,205]. (iv) The combination of a Mettl3-inhibitor with sh-circQSOX1 and anti-CTLA4 showed a promising strategy to overcome Treg cell-mediated resistance in CRC immunotherapy [146]. (v) YTHDF1-inhibition by CRISPR-Cas and VNPs-siYTHDF1 improve anti-PD1 therapeutic efficacy and overcame anti-PD1 resistance in MSS-colorectal carcinoma (CRC) [205]. (vi) Dendritic cell inhibiting YTHDF1 enhanced anti-PD1 therapeutic efficacy by increasing DC-mediated anti-tumor immunity via protecting antigen degradation and cross presentation to CD8^+^T-cells [166,167]. (vii) Macrophage (MΦ) expressing Mettl-3 improve anti-PD1 therapeutic efficacy and control lung metastasis by YTHDF1-mediated translation of Spred2 [173]. (viii) In non-small cell lung cancer (NSCLC) inhibition of Mettl-3 (STM2457) improves the therapeutic potential of checkpoint blockade therapy by upregulating PD-L1 expression [206]. (ix) In melanoma, an m^6^A-eraser protein, FTO has found to be highly upregulated and showed decreased response to anti-PD1 therapy due to YTHDF2-mediated mRNA degradation of PD-1, CXCR4 and SOX10 genes. Therefore, inhibiting FTO could overcome anti-PD1 resistance [53]. (x) ALKBH5 (an m^6^A eraser protein)-deletion in tumor cells improves the therapeutic efficacy of anti-PD1 in controlling tumorigenesis of melanoma and colorectal cancer by changing the lactate content in the tumor microenvironment by modulating MDSCs and T-regs population [207]. (xi) YTHDF1-inhibition combined with methionine restricted diet (MRD) increased anti-tumor efficacy of anti-PD1 therapy by increasing the infiltration of CD8^+^T-cells in a mouse tumor model established using the CT26 cell line [208]. These findings clearly revealed the immense potential of co-targeting m^6^A-modification machineries in improving the therapeutic efficacy of anti-PD1 in enhancing anti-tumor immunity including other immune associated disorders.

### Advancing m^6^A-regulators & clinical trials by industry developed compounds (Table 2, Table 3)

#### STC-15

STC-15 is developed by *STORM therapeutics* is a first-in-class orally available selective inhibitor of ‘Mettl-3′ scheduled phase-I clinical trial (NCT05584111) to check the safety and pharmacology against advanced malignancies [143]. Some other m^6^A-based pharmacological inhibitors in pipeline are BBB+/-ve 2nd Gen Mettl-3, Mettl-1 and ADARI to treat haematological cancer, solid tumors, Alzheimer’s, inflammation, viruses and other diseases [[209], [210], [211], [212]]. A patent (WO2020201773A1) has been filed by Storm Therapeutics [131,[136], [137], [138]] and Accent Therapeutics [[133], [134], [135]] on a compound inhibiting ‘Mettl-3 activity’ with potential applications in cancer treatment and other diseases.

#### DHX9 and KIF18A inhibitors

DHX9 inhibitor and KIF18A inhibitor are two RNA-modifying drugs currently under pipeline being developed by *Accent therapeutics*. DHX9 inhibitor target BRCA-deficient breast cancer, works on the mechanism of exploiting transcription, replication, RNA splicing, and genomic stabilities leading to death of the tumor cells. KIF18A, a mitotic kinesin, is essential for cell division, particularly in chromosomally unstable TNBC and ovarian cancer cells. Its selective inhibition by KIF18A inhibitor halts tumor cell proliferation. Recently, Accent therapeutics scheduled Phase-I/II clinical trial (NCT06625515) targeting ATX-559 (an oral inhibitor of DHX) against advanced or metastatic solid tumors. Recently, ATX-559 has received FDA fast track designation, enabling accelerated development and review in recognition of the serious conditions it addresses and the significant unmet medical need. Accent therapeutics is also working in partnership with AstraZeneca on Mettl-3 and ADAR1 to treat AML and other solid cancer (https://accenttx.com).

#### PARG inhibitors

PARG inhibitor (ETX-19477), Mettl-3, QPCT/L and ADAR1 inhibitors are the key mRNA-modifying products developed by *Gotham Therapeutics* (*8five8 Therapeutics*). PARG inhibitor is currently under phase-I clinical trial (NCT06395519) against advanced solid malignancies (ERADIC8). It works on the mechanism of inhibiting proliferation and arrest cells in the S or G2 phase and induce apoptosis in cancer cells (https://8five8tx.com).

#### EP102 and EP282

EP102 is an Mettl-3 inhibitor developed by *Epics Therapeutics* undergoing pre-clinical trial and has shown strong in vivo efficacy when administered in combination with Venetoclax (a drug targeting BCL-2) for the treatment of AML [213,214]. **EP282** is another first-in-class small molecule is an FFAR2-agonist (available in oral formulation) conducted phase-I clinical trial in January 2022 for the treatment of gastrointestinal inflammatory disease and other solid tumors.

#### Some other pharmacological inhibitors targeting

RNA-modification enzymes for example CLK, TUT4/TUT7, cGAS, and PAPD5/PAPD7 inhibitors are being developed by Twentyeight-Seven therapeutics. It works on RNA splicing, uridylation and degradation mechanisms for the treatment of inflammatory, HBV and telomere-related diseases (https://redonatx.com/pipeline). SF3B1: SF3B1 is small molecule modulator of splicing factor 3B1 (SF3B1) being developed by H3 biomedicine that regulate RNA splicing machineries. This is under phase-I clinical trial (NCT02841540) to treat haematological cancers [215,216]. Another RNA-modifying drugs like eIF4E (an oncogenic RNA-binding protein) and ‘c-MYC’ is being developed by Ribometrix Therapeutics to treat several cancers [217]. SKY-0515: Is first-in-class phase-I clinical candidate (ACTRN12624000602527) being developed by Skyhawk therapeutics. It is a small-molecule RNA-modifying drugs working on the mechanism of RNA splicing factor used to treat Huntington's disease (HD), and rare genetic conditions including cancer and neurological disorders. SkySTAR™ (Skyhawk small molecule therapeutics for alternative splicing of RNA) is their proprietary platform which designs drugs to correct pathogenic RNA splicing errors (https://www.skyhawktx.com). KRRO-110: Is the lead product of Korro Bio which is used to treat alpha-1 antitrypsin deficiency (AATD) by editing ‘Serpin-A1′ gene mutation in RNA. Korro Bio is specialised in developing new class of genetic medicine using their proprietary technology platform called OPERA (Oligonucleotide promoted editing of RNA) which aims to create precise edits in RNA through a single base change [218]. ADAR1 is another RNA editing candidates being develop in collaboration with Novo Nordisk to treat several diseases (https://www.korrobio.com/). WVE-006: Is the lead product of Wave life Sciences currently under phase-I clinical trial (NCT06405633) to treat AATD. It works on RNA editing and splicing mechanism and is used to treat lung and liver disease with genetic disorder caused by a G-to-A point mutation in the Serpin-A1 gene, also known as Z-allele [219]. AX-0810 and AX-1412: Is the lead product of ProQR Therapeutics working on their propriety Axiomer™ RNA editing technology. AX-0810 target NTCP (SLC10A1 gene) for cholestatic liver conditions and AX-1412 aimed at B4GALT1 for cardiovascular disorders (https://www.proqr.com). Some other institutions or laboratories working in collaboration with cell therapy companies in developing RNA modifying drugs are mentioned in these articles [12,220].

## Conclusion and future prospectives

In this review, we have examined the epigenetic modifications of RNA and their impact on immune cell functions. Our findings highlight the pivotal roles of RNA modification machineries (writers, erasers, and readers) in regulating immune cell biology and their potential implications in advancing immunotherapeutic strategies. m^6^A-modification is highly abundant mRNA modification among 172 identified types and is predominantly enriched near the 5′-UTR start codon, 3′-UTR stop codon, and within the coding region of the mRNA (Fig. 1**C**) underscoring its critical role in regulating not only gene expression but also governing the fate of the cells expressing these modified mRNAs. Therefore, exploring m^6^A-mRNA modification open a new scope in advancing personalized therapy co-targeting other intracellular checkpoint genes (CISH/SOCS) and checkpoint blockade (anti-PD1) antibodies.

Cancer immunotherapy is rapidly expanding filed in the treatment of haematological cancer and solid tumors. However, immune escape-like mechanisms, limited migration, lower persistence, insufficient anti-tumor responses and resistance-like issues are the major upcoming challenges. Recent advancements have demonstrated that the pharmacological inhibition of m^6^A-modification enzymes by small molecule inhibitors of Mettl-3 (STM2457, STM3006) has significantly enhanced the anti-tumor activity of CD8^+^T-cells through the formation of dsRNA, and activation of interferon signalling. Additional studies further support the therapeutic potential of Mettl3-inhibition in enhancing anti-tumor immunity. For instance, (i) the Mettl-3 inhibitor (STM2457) has been shown to mitigate TAM-mediated immunosuppression in colorectal cancer [142], (ii) Mettl-3 inhibitor (STM2743) enhances anti-tumor immunity against CRC by reducing MDSCs accumulation while promoting CD4^+^T-cells and CD8^+^T-cell proliferation [221]. A promising preclinical study (NCT05584111) has revealed the enhanced efficacy of Mettl-3 inhibition in treating haematological malignancies, especially acute myeloid leukemia (Fig. 2**G**). **STC-15** is first-in-class selective inhibitor of Mettl-3 scheduled phase-I clinical trial to evaluate its pharmacological efficacy and specificity against advanced malignancies by STORM Therapeutics.

Besides the Mettl-3 inhibitor (STM2457) in clinical development, YTHDF2 inhibitors (DF-A7, DC-Y13) have also demonstrated enhanced anti-tumor efficacy across various cancers (Table 1, Table 3). YTHDF2 knockdown has also been shown in reducing DLBCL proliferation [157]. Moreover, co-therapy targeting Mettl3-inhibitor in combination with anti-PD1 checkpoint blockade therapy further enhanced the anti-tumor activity of both CD8^+^T-cells as well as efficacy of anti-PD1 therapy against HCC and TNBCs. Selective inhibition of an m^6^A-reader protein ‘YTHDF1′ in dendritic cells (YTHDF1^cKO^) has also showed increased DC-response by increasing antigen cross-presentation. Dual therapy co-targeting checkpoint blockade antibodies and YTHDF1-inhibition showed enhanced DC-mediated anti-tumor immunity and improved anti-PD1 efficacy (Fig. 4**B**). Mettl3-expressing macrophage (MΦ) showed enhanced anti-tumor immunity against lung metastasis by recruiting YTHDF1 reader proteins. Moreover, co-therapy targeting Mettl-3 engineered macrophage showed improved efficacy of anti-PD1 therapy (Fig. 4**D**). These findings clearly suggest the immense potential of co-targeting intracellular m^6^A-modifiers in augmenting the anti-tumor activity of immune cells within a combination therapy strategy.

Interestingly, Mettl-3 inhibitors (STM2457) present a double-edged sword in cancer therapy by enhancing CD8^+^T-cell function (Fig. 2**G**) while potentially impairing NK-cell cytotoxicity (Fig. 3**C**). This trade-off's impact likely varies by cancer type and patient factors. Optimizing dose and exploring combination therapies may help balance these effects. Further research is crucial to maximize benefits and minimize drawbacks in different cancer contexts.

Allograft survival and high-dose immunosuppressive medication is one of the biggest concerns in organ transplantation. Targeting m^6^A-modifiers in combination with other immunosuppressive drugs has shown significant progress in prolonging allograft survival. Hyperactivation of CD4^+^T-cell effector function is the main cause of allograft rejection; however, Mettl-3 inhibition (STM2457) showed reduced CD4^+^T-cell effector program and prolong allograft survival (Fig. 2**D**). Dendritic cell ‘activation’ is another cause of cardiac rejection in organ transplantation. Inhibiting Mettl-3 in dendritic cells (siMettl-3) showed decreases DC-activation and prolong allograft survival in a mouse heart transplant model (Fig. 4**C**). Mettl-14 inhibition in regulatory T-cells (Foxp3-Mettl14^f/f^cKO) impair T-reg suppressive functions resulted in the rejection of islets allograft by regulating SOCS family of proteins, however, Mettl-14 engineered T-regs prevented allograft rejection (Fig. 2**F**). These findings clearly revealed the potential of targeting m^6^A-modifiers in transplantation surgery.

Autoimmune disease is another major issue limiting the therapeutic efficacies of current medication. The hyperactivation of CD4^+^T-cells and downregulated Mettl3-level is the major cause of SLE in patient with autoimmune disorder. However, Mettl-3 engineered CD4^+^T-cell showed decreased activation and reduced lupus-like phenotypes by m^6^A-mediated rescue of regulatory T-cell differentiation program (Fig. 2**C**). Increased CD4^+^T-cell pathogenicity and upregulated level of an m^6^A-demethylase ‘ALKBH5′ is another cause of aggravated autoimmune disease in an animal model of brain inflammation (EAE). CD4^+^T cell-specific deletion of ALKBH5 (ALKBH5^f/f^CD4-CRE) mice showed reduced CD4^+^T-cell pathogenicity and thus decreased autoimmune response (Fig. 2**E**). These findings further highlight the potential of co-targeting m^6^A-modifiers in controlling autoimmune disorders.

The intracellular checkpoint gene CISH (cytokine-inducible SH2-containing protein) also known as the ‘master regulator of cytokine signalling’ is the eighth member of the SOCS (suppressor of cytokine signalling) family of proteins, has garnered significant attention in improving the efficacy of immunotherapeutics. For instance, Mettl3-mediated CISH regulation is critical for maintaining CD4^+^T-cell homeostasis (Fig. 2**A**), modulating T_FH_ cell-mediated humoral immunity (Fig. 2**H**), and controlling regulatory T cell-mediated inflammatory responses (Fig. 3**A**). Similarly, Mettl14-targeted SOCS1-3 plays a role in Treg-mediated allograft transplantation (Fig. 2**F**), FTO-targeted CISH and SOCS3 in NK cell-mediated anti-tumor immunity (Fig. 3**D**), and YTHDF2-targeted SOCS3 in MDSCs-mediated anti-tumor immunity (Table 1, Table 3). These findings clearly revealed the potential of CISH in improving immune cell functions. Recently, the U.S. FDA approved AMTAGVI (Lifileucel) a CISH-targeted tumor-infiltrating lymphocyte (TIL)-based immunotherapy to treat advanced melanoma, developed by Intima Biosciences (NCT04426669) [222,223]. Furthermore, companies such as Nkarta Therapeutics [224], ONK Therapeutics, Fate therapeutics and Asher Bio are also leveraging CISH targeting to develop cell-based therapeutics, underscoring the growing clinical relevance of CISH in advancing immunotherapy.

Despite the clinical success of Kymriah and Yescarta challenges such as limited CAR T-cell efficacy and relapsed cases of leukemia and multiple myeloma remain significant hurdles. Co-therapy targeting intracellular m^6^A-modifiers offers a promising strategy to enhance CAR T-cell effector programs, addressing critical limitations such as persistence and off-the-shelf life. Moreover, the intracellular inhibition of Mettl-3 combined with CD19-CAR T-cell therapy could produce a synergistic effect in the treatment of DLBCL [155]. Furthermore, targeting m^6^A-modifiers holds great potential for the biopharmaceutical industries in manufacturing and quality development of biosimilar products. Taken together, m^6^A-modifiers present a transformative opportunity to advance immunotherapy in clinical settings and drive innovation in academia and biopharmaceutical research. The future is not so far where targeted therapies combined with epigenetic drugs and immunotherapy, could provide a universal solution to treat many diseases.

## Consent for publication

All authors have approved for the publication of the manuscript in the Journal of Advanced Research.

Data availability

Not applicable.

## CRediT authorship contribution statement

**Sunil Kumar:** Conceptualization, Investigation, Writing – original draft, Writing – review & editing, Visualization. **Mithun Sinha:** Investigation, Writing – review & editing, Visualization, Supervision, Project administration, Funding acquisition.

## Ethics approval and consent to participate

Not applicable.

## Funding

This work was partly supported by US NIH grants R01AI165958 and R21AI171932 to MS and Plastic Surgery Foundation grant 831,458 to MS. The content is solely the responsibility of the authors and does not necessarily represent the official views of the NIH or other sponsors.

## Declaration of competing interest

The authors declare that they have no known competing financial interests or personal relationships that could have appeared to influence the work reported in this paper.
